# Synthetic vaccine particles for durable cytolytic T lymphocyte responses and anti-tumor immunotherapy

**DOI:** 10.1371/journal.pone.0197694

**Published:** 2018-06-01

**Authors:** Petr O. Ilyinskii, Grigoriy I. Kovalev, Conlin P. O’Neil, Christopher J. Roy, Alicia M. Michaud, Natalia M. Drefs, Mikhail A. Pechenkin, Fen-ni Fu, Lloyd P. M. Johnston, Dmitry A. Ovchinnikov, Takashi Kei Kishimoto

**Affiliations:** 1 Selecta Biosciences, Watertown, MA, United States of America; 2 SelectaRUS, Khimki, Moskovskaya oblast, Russia; Mie University Graduate School of Medicine, JAPAN

## Abstract

We previously reported that synthetic vaccine particles (SVP) encapsulating antigens and TLR agonists resulted in augmentation of immune responses with minimal production of systemic inflammatory cytokines. Here we evaluated two different polymer formulations of SVP-encapsulated antigens and tested their ability to induce cytolytic T lymphocytes (CTL) in combination with SVP-encapsulated adjuvants. One formulation led to efficient antigen processing and cross-presentation, rapid and sustained CTL activity, and expansion of CD8^+^ T cell effector memory cells locally and centrally, which persisted for at least 1–2 years after a single immunization. SVP therapeutic dosing resulted in suppression of tumor growth and a substantial delay in mortality in several syngeneic mouse cancer models. Treatment with checkpoint inhibitors and/or cytotoxic drugs, while suboptimal on their own, showed considerable synergy with SVP immunization. SVP encapsulation of endosomal TLR agonists provided superior CTL induction, therapeutic benefit and/or improved safety profile compared to free adjuvants. SVP vaccines encapsulating mutated HPV-16 E7 and E6/E7 recombinant proteins led to induction of broad CTL activity and strong inhibition of TC-1 tumor growth, even when administered therapeutically 13–14 days after tumor inoculation in animals bearing palpable tumors. A pilot study in non-human primates showed that SVP-encapsulated E7/E6 adjuvanted with SVP-encapsulated poly(I:C) led to robust induction of antigen-specific T and B cell responses.

## Introduction

Immunotherapy has become one of the most promising fields in cancer research, with checkpoint inhibitors becoming a standard of care against several types of cancer [1–3] and engineered T cells scoring successes in early clinical trials [3–5]. However cancer vaccines have been a notable exception, with a number of late stage clinical trial failures [6–8]. There are three key hurdles facing cancer vaccine development: 1) since tumors are derived from normal cells, most tumor antigens are poorly immunogenic self-proteins; 2) while prophylactic vaccines are primarily directed at inducing an antibody response, an effective therapeutic cancer vaccine must elicit a strong cytolytic T cell response, which, in turn, requires efficient cross-presentation of antigen to naïve T cells; and 3) tumors create an immunosuppressive environment that may inhibit T cell activation [9–12].

The issue of low immunogenicity for a particular antigen may be partially overcome by nanoparticle (NP) encapsulation [13–15]. Multiple groups, including ours, have shown that the encapsulation of whole protein in NP carriers significantly augments the immune response due to targeted delivery of protein to antigen-presenting cells (APCs) leading to improved antigen processing and more efficient cross-presentation [14–17] (see recent reviews in [18–20]). Additionally, co-encapsulation of strong adjuvants (e.g., toll-like receptor (TLR) agonists) further enhances immunogenicity of NP-encapsulated antigens in a manner that mitigates systemic cytokine production [15]. We have previously demonstrated that TLR agonists encapsulated in Synthetic Vaccine Particles (SVP), stimulate focused, local immune responses in the draining lymph node without inducing production of systemic inflammatory cytokines [15].

One strategy to minimize antigen risk in evaluating cancer vaccine technologies is to target human papilloma virus (HPV)-associated neoplasms, using well characterized viral oncogenes, E6 and E7 [21]. HPV is an etiological agent for cervical carcinoma and other anogenital malignancies and is also linked to over 50% of cases of oropharyngeal cancers in the U.S. and Northern Europe. Worldwide cervical cancer incidence is approximately a half million cases [22, 23], with over 4000 deaths attributable to cervical cancer in the US each year [24]. There is an unmet need for more effective (and less invasive) immunotherapeutic approaches. A number of therapeutic vaccine strategies including live vector, peptide, protein, DNA, RNA replicon, and dendritic cell based vaccines targeting HPV E6 and E7 [25] are currently being investigated [26–40].

Here we describe development of a SVP formulation with a high potential of inducing efficient and sustained CTL responses and strong therapeutic anti-tumor responses in vivo. Our objective was to optimize tumor vaccines by assessing different polymer formulations, TLR agonists, tumor antigens, and synergy with checkpoint inhibitors and chemotherapeutic agents. The lead SVP-based vaccine candidate encapsulating HPV-16 E7/E6 fusion protein and endosomal TLR agonists demonstrated high immunogenicity and durable immune memory in vivo, which lead to efficient tumor control against the HPV-16 E6- and E7-expressing TC-1 cell line, even when therapy was delayed until tumors were palpable. Moreover, SVP-based vaccines acted synergistically with checkpoint inhibitors, such as anti-PD-L1 antibodies, and also with cisplatin, the principal chemotherapeutic agent used in cervical cancer therapy. Finally, we have translated key findings in a pilot study in nonhuman primates (NHP) to assess the immunogenicity of an SVP HPV vaccine. Comparative evaluation of three SVP-encapsulated endosomal TLR agonists, R848, CpG ODNs and poly(I:C), revealed that while all three supported robust CTL induction in mice, the TLR3 agonist, poly(I:C), was superior to TLR7/8 or TLR9 agonists in NHP.

## Materials and methods

### Preparation of nanoparticles

The nanoparticles were manufactured using either a single emulsion or double emulsion method as described [15, 41] or indicated in the Supporting information Material and Methods (S1 Methods).

### Mice

Six- to eight-week-old female C57BL/6 mice were purchased from Charles River Laboratories (Wilmington, MA, USA), Taconic (Germantown, NY, USA) or Nursery for Laboratory Animals of Institute of Bioorganic Chemistry of the Russian Academy of Science (Moscow, Russia). All animal protocols were reviewed and approved by institutional IACUCs in accordance with federal (USA or Russia) and state (MA) regulations and guidelines (Selecta Biosciences Protocol Number: SBI2015-003, Institute of Bioorganic Chemistry Protocol numbers 152/2014 and 203/2016).

### In vivo tumor inoculation

Cells were injected s.c. (intrascapular, 5×10^4^/mouse). Mice were monitored daily for health status and 2–3 times a week for tumor progression. The tumor volume determination was performed by external caliper measurement of length and width of tumor and calculation of the volume by use of the modified ellipsoid formula - ½(Length × Width^2^). Animals were euthanized if one of the measurements exceeded 20 mm or if the animal became moribund. Specifically, humane endpoints were used in all tumor studies (death as an endpoint was never utilized). In addition to tumor measurements being taken 2–3 times a week by trained scientific personnel and animals being euthanized if any measurement exceeded 20 mm (or if an animal was judged as moribund by phenotypical features), all mice have been health-checked by a trained animal technician on a daily basis and if judged moribund, euthanized during the same day (<8 hours). Moreover, in all the studies involving TC-1 cell line, all mice were weighed prior to and during the experiment and the weight loss of >15% was used as another criterion for euthanasia (EG.7-OVA and B16-F10 lines are not known to cause a weight loss within defined tumor size and they never did in any of studies reported herein). No animals died before meeting euthanasia criteria with exception of extremely rare events not exceeding spontaneous mortality rate in laboratory mice of the same age and strain. Duration of each experiment is shown in each figure in Results section, but overall short-term experiments involving aggressive tumor cell lines or SVP of limited immunogenicity lasted 45–80 days, while long-term experiments using TC-1 cell line and highly immunogenic SVP lasted 100–500 days. All short-term tumor treatment experiments used 5 mice per group in a single study, while long-term TC-1 studies involved 7–8 mice per group with their mortality rate shown in corresponding figures. All euthanasia procedures were performed by cervical dislocation under isoflurane anesthesia according to AVMA Guidelines for the euthanasia of animals (Section 2.2). Most animals surviving tumor challenge were not euthanized at the end of the study, but kept under daily observation and used later in long-term immune memory studies as described in Results.

### Vaccination

Mice were injected s.c. in both hind limbs (30 μl volume per injection site, 60 μl total) with PBS vehicle containing SVP-formulated or free antigens and adjuvants. A single time-point injection was used in cytokine production and in vivo CTL assay experiments, and prime-boost regimens starting at d3-14 post tumor inoculation with the 1^st^ boost on day 3 or 4 after the prime followed by two additional weekly boosts were used when assessing anti-tumor efficiency.

### Combination treatments

In studies to assess synergy with checkpoint inhibitors, mice received SVP on days 14, 17, 24 and 31 and monoclonal antibodies to PD1 (250 μg), PD-L1 (200 μg) or rat IgG2b isotype (250 μg) i.p. on days 18, 21 and 25 for TC-1 tumors or SVP on days 3, 7, 14 and 21 and anti-CTLA 4 (200 μg), anti-PD1 (250 μg/mouse), anti-PD-L1 (250 μg/mouse) or rat IgG2a or IgG2b isotype (Bio X Cell, West Lebanon, NH) for B16-F10 tumors. In studies to assess synergy with chemotherapy, mice were injected with 5 mg/kg cisplatin i.p. on days 5 and 12 after TC-1 cell inoculation followed by SVP immunizations starting on day 14 or 21.

### In vivo cytotoxicity

Antigen-specific cytolytic activity in vivo was determined as described [15] at different time-points after a single SVP immunization. Other immunological readouts are described in detail in the Supporting information Materials and Methods (S1 Methods).

### NHP studies

All procedures in NHP study were in full compliance with the U.S. Department of Agriculture’s (USDA) Animal Welfare Act (9 CFR Parts 1, 2, and 3); the Guide for the Care and Use of Laboratory Animals and the National Institutes of Health, Office of Laboratory Animal Welfare. Whenever possible, procedures were designed to avoid or minimize discomfort, distress, and pain to animals. *Macaca fascicularis* (cynomolgus macaque) females, Mauritius origin, weight of 3.7–5.1 kg were used (Covance, Inc., Princeton, NJ) in accordance with Covance Research Products standard operating procedures, protocol PA 0006–16 and amendments. Animals were kept in stainless steel cages (with enrichment and socialization) on Purina Diet 5049, fed according to Testing Facility SOP with water available ad libitum. Animals were acclimated for 18 days prior to pre-blood collection on study day -23. SVP vaccines were injected s.c. in 1 mL in two sites in quadriceps muscles area on days 0, 21 and 42. To minimize discomfort, distress, and pain each primate received 10 mg/kg of ketamine at 100 mg/ml intramuscularly prior to vaccine administration. During the study animals were observed daily for signs of morbidity and/or mortality and body weights were measured on days -23, -16, 0, 14, 35, 49, 56 and 70. PBMC samples were isolated from heparinized blood were used for ELISPOT, CTL cytotoxicity and FACS analyses, as detailed in the Supporting information Materials and Methods. Serum was used in ELISA to test for antibodies to HPV-16 E7 and E6. At the end of the experiment animals were euthanized by exsanguination under general anesthesia by Ketaset Primate according to AVMA guidelines.

## Results

### Nanoparticle encapsulation of model antigen in PLGA leads to induction of durable effector CD8^+^ CTL responses and promotes sustained anti-cancer activity in vivo

**Fig 1:**
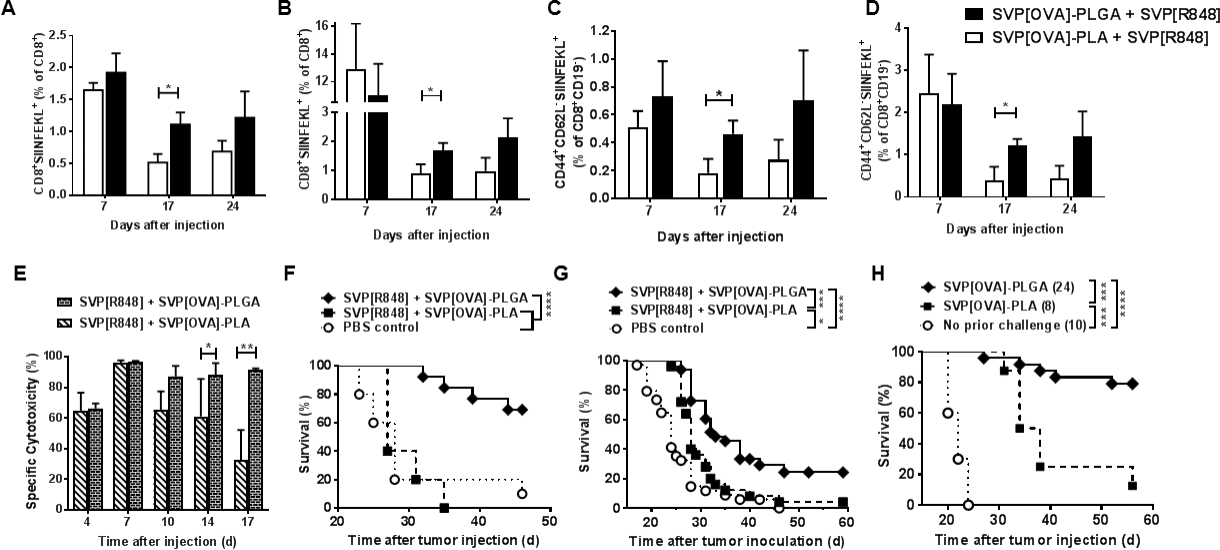
CTL induction and anti-tumor activity of two SVP formulations. A-D. Analysis of T lymphocyte populations after immunization with SVP. Animals (4 mice/group/time-point) have been injected with SVP and antigen-specific T cells evaluated. A, B–cells from draining lymph nodes (A) or spleens (B) stained for CD8 and SIINFEKL-specific TCR (CD19^+^ cells gated out). C, D. CD8^+^SIINFEKL-specifc cells are stained for CD62L and CD44 T markers. C–popliteal lymph nodes; D–spleens. Summary of two independent experiments is shown. E. SVP induction of antigen-specific cytotoxicity. Animals (3–6 per time-point in each group) were injected with SVP[OVA]-PLA or SVP[OVA]-PLGA combined with SVP[R848] and CTL activity measured in vivo at times indicated. F-H. Anti-tumor effect of SVP immunization. Animals inoculated with EG.7-OVA cells were treated with SVP[OVA]-PLA or SVP[OVA]-PLGA combined with SVP[R848] at days 1, 4, 11, and 18 (F) or 3, 7, 14, and 21 (G) by s.c. administration at a tumor-distant site. H. SVP-treated animals surviving EG.7-OVA challenge were re-challenged with the same cells without additional treatment. Summary of two (F, H) or five (G) independent experiments is shown. * p <0.05, ** p <0.01, *** p<0.001, **** p<0.0001.

Two SVP formulations of ovalbumin (OVA), SVP[OVA]-PLA and SVP[OVA]-PLGA, were tested in combination with SVP-encapsulated TLR7/8 agonist R848 (SVP[R848]) for their ability to induce antigen-specific CD8 T cells in vivo. While both formulations induced similar levels of CD8 T cells directed against the dominant MHC class I OVA peptide (OVA_257-264_ or OP.I) at 7 days after administration, high levels of antigen-specific CD8 T cells persisted for at least 24 days in draining lymph nodes and the spleen after a single administration SVP[OVA]-PLGA (Fig 1A and 1B). Further analysis showed that the SVP[OVA]-PLGA formulation induced a significant increase in the percentage of effector memory T cells (CD62L^low^CD44^+^) in the spleen (>3-fold) and in local lymph nodes (>2.5-fold) compared to the SVP[OVA]-PLA formulation (Fig 1C and 1D). No difference was noted between the SVP formulations in the induction of central memory T cell populations (CD62L^high^CD44^+^).

Both SVP formulations induced similarly potent CTL activity in vivo at 4–7 days after administration, with nearly complete target elimination seen at day 7 (Fig 1E). However, the level of CTL activity induced by SVP[OVA]-PLA declined by day 10 and continued to decrease through day 17. In contrast, the CTL activity induced by SVP[OVA]-PLGA was sustained, with >90% elimination of target cells through day 17 (Fig 1E) and >30% activity at day 31 after a single SVP injection (S1 Fig).

SVP[OVA]-PLGA was similarly superior to SVP[OVA]-PLA when used in combination with SVP[R848] for therapeutic treatment of EG.7-OVA tumors (Fig 1F and 1G). This was especially pronounced if surviving animals were re-challenged with the same tumors without any additional treatment (Fig 1H). In this case, the majority of animals previously treated with SVP[OVA]-PLGA and SVP[R848] survived the repeat challenge, while most animals previously treated with SVP[OVA]-PLA and SVP[R848] did not. These results may reflect the generation of a more effective memory response induced by SVP[OVA]-PLGA, as seen in Fig 1C and 1D. Notably, EG.7-OVA tumors from animals treated with SVP[OVA]-PLGA and SVP[R848] which escaped immune surveillance and elimination showed loss of the transduced OVA gene with only one out of six samples being weakly positive for OVA, while six out six tumors from mock-treated mice were all strongly OVA-positive (S2 Fig).

Cellular distribution of SVP following s.c. administration was analyzed using SVP-PLA or SVP-PLGA particles formulated with fluorescent Cy5-labeled polymer and self-quenched DQ-OVA (OVA which emits fluorescence upon proteolysis, [42]). No differences in uptake of SVP[DQ-OVA]-PLA versus SVP[DQ-OVA]-PLGA were seen among B cells, plasmacytoid DC, myeloid DC, and conventional DC (as measured by Cy5 fluorescence, S3A–S3E Fig, top row). Similarly no difference in ovalbumin processing (measured by DQ fluorescence) was observed within B cells that had endocytosed SVP[DQ-OVA]-PLA versus SVP[DQ-OVA]-PLGA (S3A Fig, bottom row). However, all DC subtypes within the draining LN showed more extensive proteolytic processing of DQ-OVA when formulated within SVP-PLGA, especially at a 24-hour time-point (S3B–S3D Fig, bottom row). Better processing of OVA formulated within SVP-PLGA may account for the superior induction of effector CD8 T cells observed in Fig 1.

In additional studies, SVP[OVA]-PLGA also demonstrated an ability to induce antibody responses to OVA (S4 Fig, note that from this point on, only PLGA-based SVP formulations were used to encapsulate test antigens). In particular, three immunizations of SVP[OVA] adjuvanted with SVP-encapsulated TLR agonists were capable of maintaining high anti-OVA antibody titers for 1–2 years after the initial immunization (S4A and S4B Fig). At this point, SVP-encapsulated CpG oligonucleotides (ODN), potent TLR9 agonists, were tested and compared to SVP[R848]. Natural CpG ODNs contain a phosphodiester (PO) backbone (PO-CpG), which is susceptible to rapid hydrolytic cleavage by nucleases in vivo. Nuclease-resistant CpG sequences with a phosphorothioate (PS) backbone (PS-CpG) have been shown to have superior activity to PO-CpG in vivo and have been utilized in many preclinical and clinical studies [43]. Notably, similar levels of antibody to OVA were produced when SVP[OVA] was combined with either SVP-encapsulated PS- or PO-CpG ODNs, exceeding titers induced by SVP[OVA] alone by a factor of 7–17 when measured nearly a year after the last immunization (S4B Fig). These results indicate that SVP encapsulation of PO-CpG shields the oligonucleotide from nucleases and enables effective delivery to antigen-presenting cells.

Moreover, mice immunized with SVP[OVA] with adjuvant on days 0, 21, and 42 and then boosted 700 days after the last treatment with SVP[OVA] and SVP[R848] demonstrated an excellent anamnestic antibody-producing response resulting in increases in antibody titers by a factor of 12 if only 0.1 μg of SVP-encapsulated OVA was used and as high as a factor of 35 if 10 μg of SVP-encapsulated OVA was used (S4C Fig). Similarly, CD8 T cell long-term memory recall responses were readily detectable in 57 out of 58 tested animals 200–400 days after the last SVP administration (S4D and S4E Fig).

### Therapeutic activity of SVP encapsulated peptide vaccines

**Fig 2:**
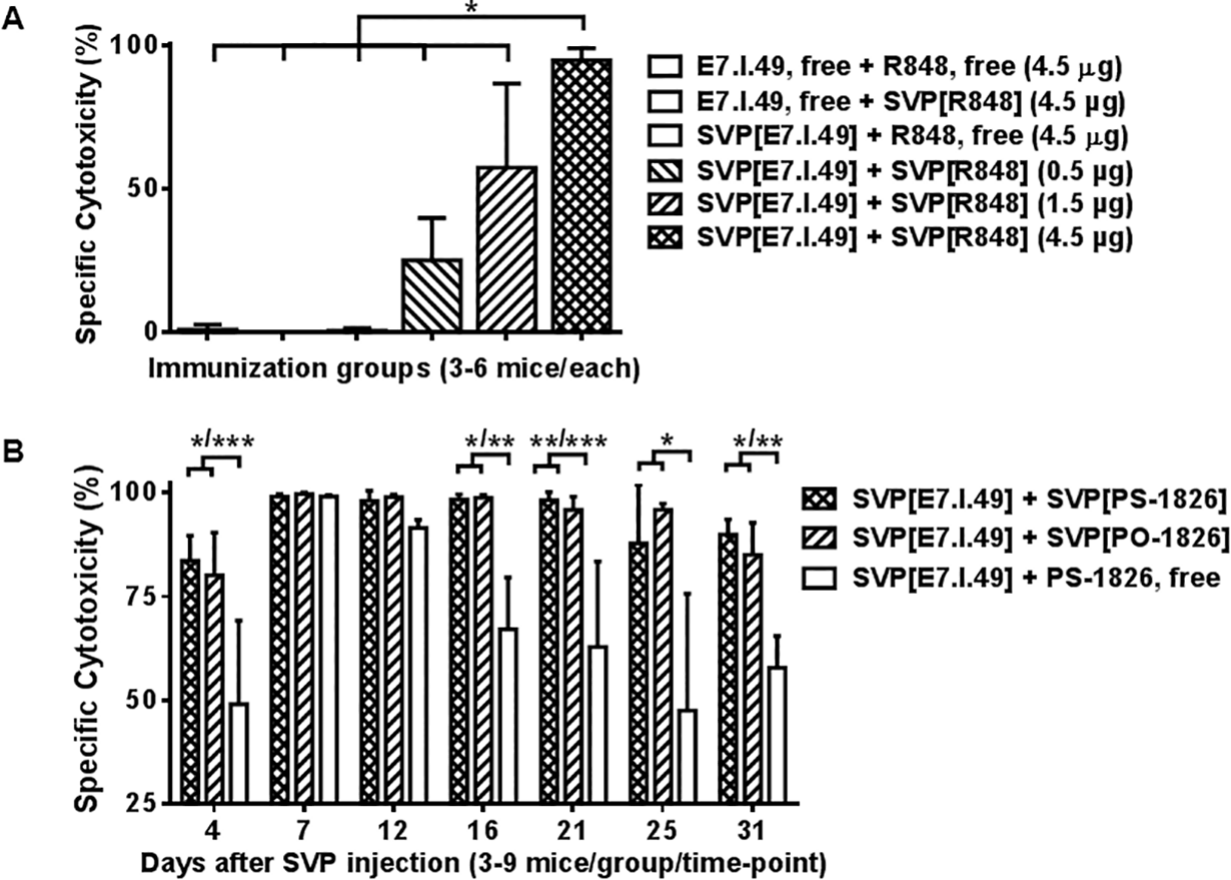
SVP-induced CTL activity against dominant CTL epitope of HPV-16 E7 protein. Mice were immunized with free or SVP-entrapped E7.I.49 peptide (3 μg) and TLR agonists and in vivo CTL activity measured. A. R848 used as adjuvant (doses indicated), assay conducted at 7 days after vaccination. B. PS- or PO- forms of CpG-1826 (4 μg) used as adjuvant. Assay dates shown. * p<0.05, ** p <0.01, *** p<0.001.

We next evaluated CTL induction against E7.I.49, the dominant MHC class I epitope of the HPV-16 E7 oncoprotein. Encapsulating both E7.I.49 and R848 within the SVP led to the dose-dependent induction of robust CTL activity, while free (non-encapsulated) E7.I.49 and R848 were inactive (Fig 2A). Next we assessed the efficacy of SVP[E7.I.49] administered with free CpG versus SVP-encapsulated CpG using the mouse-specific CpG sequence 1826. SVP[PS-CpG]- and SVP[PO-CpG]-adjuvanted vaccines induced more rapid onset of CTL activity than the free PS-CpG-adjuvanted vaccine (Fig 2B). Moreover, while CTL activity induced with the free PS-CpG-adjuvanted vaccine decreased after 7 days, the SVP[PS-CpG]- and SVP[PO-CpG]-adjuvanted vaccines induced sustained CTL activity, with nearly complete elimination of target cells observed through day 21 and 85–95% activity for at least one month. Notably, free nuclease-susceptible PO-CpG was completely inactive (not shown).

**Fig 3:**
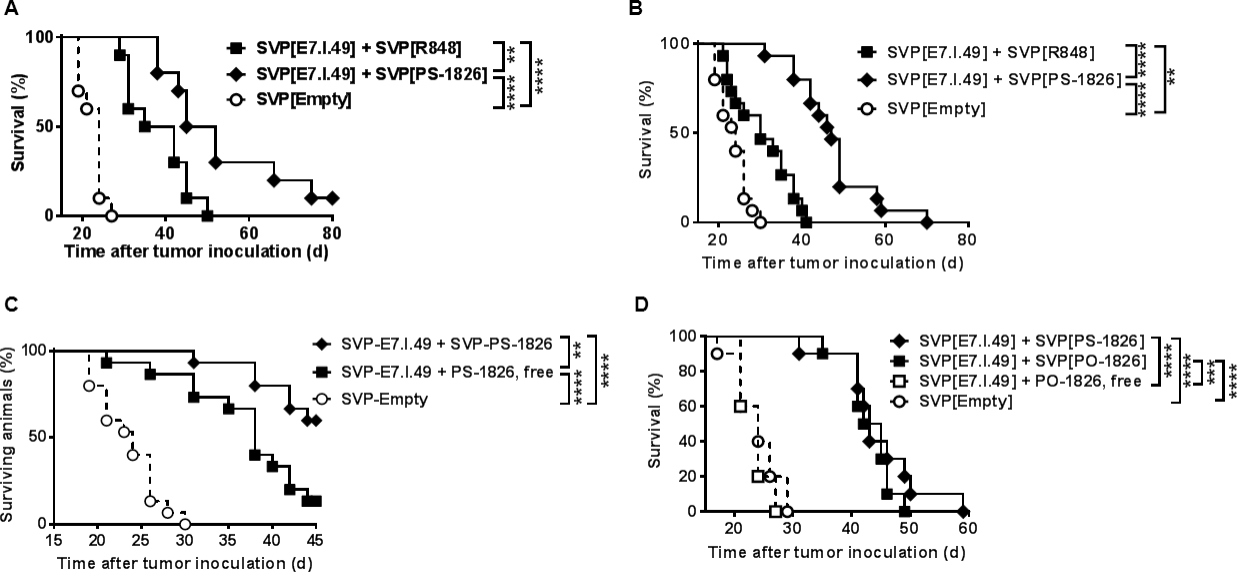
Treatment of TC-1 tumors by SVP[E7.I.49] combined with different adjuvants. Survival proportions are shown on each graph. A, B. SVP-entrapped R848 vs. PS-1826 CpG. Treatments administered on days 3, 7, 14 and 21 (A) or days 6, 10, 17 and 24 (B) after tumor inoculation. Summary of three (A) or four (B) independent experiments shown. C, D. SVP-entrapped or free CpG ODN; PS-1826 (C) or PO-1826 (D). Treatments administered on days 6, 10, 17 and 24 after tumor inoculation. Summary of three (C) or two (D) independent experiments is shown. ** p <0.01, *** p<0.001, **** p < 0.0001.

We next evaluated the efficacy of SVP[E7.I.49] against TC-1 tumors in vivo. This mouse tumor line was transduced to express the E6 and E7 oncogenic proteins of HPV-16 [44]. SVP[E7.I.49] was more effective in promoting survival in TC-1 bearing animals when adjuvanted with SVP[PS-CpG] than with SVP[R848] (Fig 3A and 3B). This difference became even more pronounced when treatment was initiated at 6 days versus 3 days after tumor inoculation (compare Fig 3B to Fig 3A). Moreover, SVP[PS-CpG] was more effective than free PS-CpG (Fig 3C). SVP-encapsulated PO- and PS-CpG were equally potent, while free PO-CpG provided no benefit (Fig 3D). Notably, injections of SVP[PO-CpG] using an intensive anti-tumor therapeutic regimen (S5A Fig) or at a more typical prophylactic vaccination schedule (S5B Fig) showed reduced local tissue inflammation compared to equivalent doses of free PS-CpG for both CpG sequences tested, 1826 and 7079.

SVP[E7.I.49] adjuvanted with SVP[R848] was also effective in an experimental TC-1 metastasis tumor model, with no metastases observed in lungs at d32 after i.v. tumor inoculation (S6A and S6B Fig).

SVP vaccines encapsulating Trp2(180–88) peptide were also efficacious in enhancing survival in mice inoculated s.c. with the B16-F10 melanoma cell line. SVP[PO-CpG] was a more effective adjuvant than SVP[R848] (S6C Fig), with the former promoting an average half-life expectancy of 10 days longer than the SVP[R848]-adjuvanted vaccine (and three weeks longer than untreated mice) and enabling long term (>80 days) survival in 20% of inoculated mice (S6C Fig).

### Therapeutic efficacy of SVP vaccines against HPV-16 oncogenic proteins in mice with established palpable TC-1 tumors

**Fig 4:**
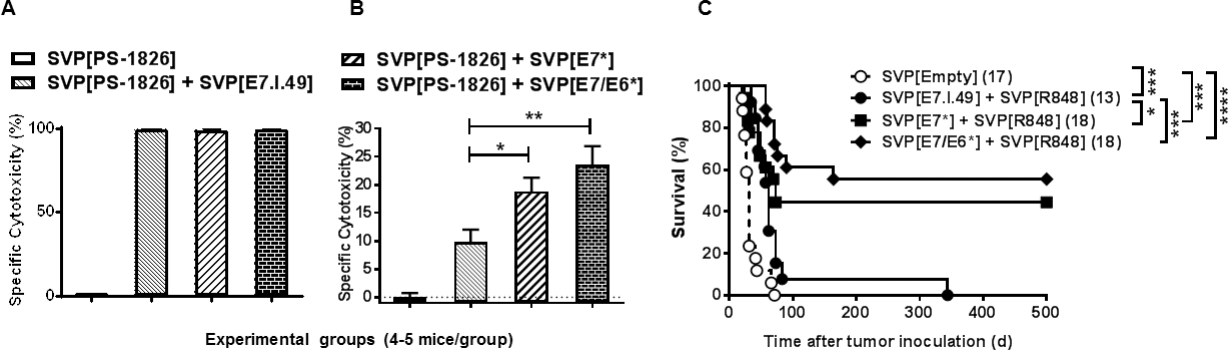
Immunogenicity of SVP-entrapped HPV-16 antigens and their therapeutic efficacy in vivo. A, B. Antigen-specific cytotoxicity at 7 days after immunization with SVP[E7.I.49], SVP[E7*] or SVP[E7/E6*]. Target cells were pulsed by E7.I.49 peptide (A) or by a pool of subdominant E7 peptides (B). C. Treatment of TC-1 tumors by SVP-entrapped E.I.49, E7* or E7/E6* combined with SVP[R848], SVP administered on days 10, 14, 21 and 28 after tumor inoculation. Number of mice in each group is shown in parentheses. Summary of four independent experiments is shown. * p <0.05, ** p < 0.01, *** p < 0.001, **** p < 0.0001.

Next we proceeded to construct protein-based HPV antigens capable of inducing broad and long-term immune response to HPV-16 oncogenic proteins. Two genetically-modified antigens, mutated E7 protein (E7*, bearing two point mutations abolishing E7 binding to p105-Rb) and a fusion of mutated E7 and E6 proteins (E7/E6*, additionally bearing mutations abolishing E6 binding to p53) were constructed, encapsulated into SVP and tested for their immunogenicity and therapeutic activity. Both SVP[E7*] and SVP[E7/E6*] were immunogenic in vivo, as assessed by their ability to induce antibody responses (S7 Fig) and cytotoxic responses to E7 (Fig 4A and 4B). Notably, immunization with SVP[E7*] and SVP[E7/E6*] induced cytolytic T cell responses against the dominant E7.I.49 peptide which was equal to that induced by SVP[E7.I.49] (Fig 4A). Moreover, the SVP[E7*] and SVP[E7/E6*] vaccines induced significantly better CTL responses against subdominant E7 epitopes than the SVP[E7.I.49] vaccine (Fig 4B).

Both SVP[E7*] and SVP[E7/E6*] showed significantly higher therapeutic activity than SVP[E7.I.49] when combined with SVP[R848] (dosing d6, 10, 17, 24 after tumor inoculation, as described in Fig 3B), resulting in long-term survival rates of 150 days in 60–70% of mice (not shown). Moreover, this difference was even more pronounced if the start of treatment was delayed until day 10 after tumor inoculation, at which point most TC-1-inoculated mice exhibited palpable tumors. In this case, SVP[E7.I.49] combined with SVP[R848] delayed tumor growth but provided minimal long-term survival advantage over the mock-treated control (Fig 4C). In contrast, treatment with SVP[E7*] or SVP[E7/E6*] led to statistically higher life-expectancy and survival rates, with 44% and 56%, respectively, of mice surviving at least 500 days (Fig 4C).

### Comparison of the therapeutic activity of SVP[E7/E6*] combined with SVP-encapsulated TLR3, TLR7/8, or TLR9 agonists

**Fig 5:**
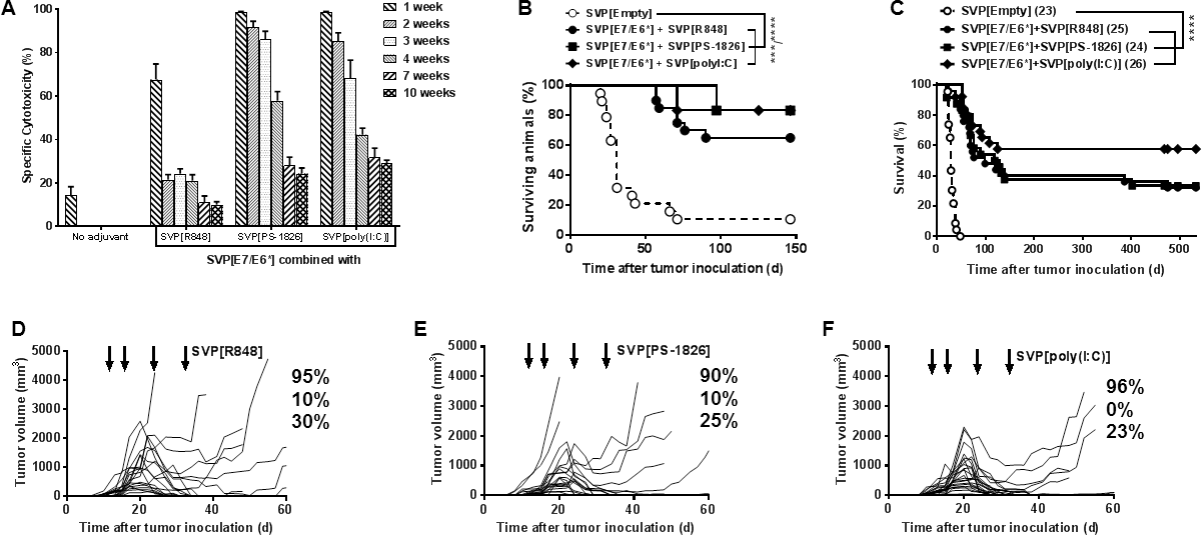
CTL induction and treatment of TC-1 tumors by SVP-encapsulated antigens and adjuvants. A. Dynamics of CTL activity after a single immunization with SVP[E6/E7*] and SVP-encapsulated adjuvants. 7–8 mice per each time-point in each group with the exception of 3 week time-point (4 mice/group). B-F. Survival (B, C) and tumor growth (D-F) in TC-1-inoculated mice treated with SVP. B, C. SVP[E7/E6*] combined with SVP-entrapped R848, CpG or poly(I:C); treatments on days 10, 14, 21 and 28 (B) or days 13/14, 17, 24 and 31 (C) after tumor inoculation. Each curve is a summary of 2 or 4 independent experiments. D-F. SVP[E7/E6*] combined with SVP[R848] (D), SVP[CpG] (E) or SVP[poly(I:C)] (F); treatments as in C (shown by arrows). The total number of mice (summary of 3 independent experiments run in parallel) is 20 in D, E and 22 in F. Percentages indicate (top to bottom) shares of mice with detectable tumors prior to treatment, those of early tumor breakthroughs and of immediate post-treatment breakthroughs.

SVP[E7/E6*] was further evaluated to compare its efficacy when paired with SVP-encapsulated poly(I:C), a TLR 3 agonist, versus SVP[R848] or SVP[CpG] (utilizing mouse-specific PS-1826). A single injection of SVP[E7/E6*] combined with SVP[R848], SVP [CpG] or SVP[poly(I:C)] induced potent in vivo CTL activity (Fig 5A). However, with SVP[R848], antigen-specific cytotoxicity exceeded 90% at day 7 in only 2 out of 8 mice, with an average activity of 68%, and then dropped precipitously by week 2 (Fig 5A). In contrast, utilization of either SVP[CpG] or SVP[poly(I:C)] led to near-complete elimination of target cells at day 7 with sustained high levels of antigen-specific cytotoxicity persisting for several more weeks before falling to 40–60% activity at 4 weeks (Fig 5A).

Next SVP[E7/E6*] was tested with the same SVP adjuvant formulations for therapeutic activity in mice with palpable TC-1 tumors, with the start of treatment delayed until 10 days after tumor inoculation. Both SVP[CpG]- and SVP[poly(I:C)]-adjuvanted vaccines led to >80% long-term survival, while SVP[R848] induced 65% long term survival (Fig 5B). When the start of treatment was further delayed until day 13 or 14 after tumor inoculation, all three SVP adjuvant formulations showed similar efficacy through 60 days (Fig 5C–5F). However, the SVP[poly(I:C)]-adjuvanted vaccine maintained a long-term survival rate of almost 60% through day 150, compared to 31% and 27% for SVP[CpG]- and SVP[R848]-adjuvanted vaccines, respectively (Fig 5C). The activity of the the SVP[poly(I:C)]-adjuvanted was also associated with a significant elevation of tumor-infiltrating lymphocytes (3.8% vs. 0.5% in mock-treated mice).

**Fig 6:**
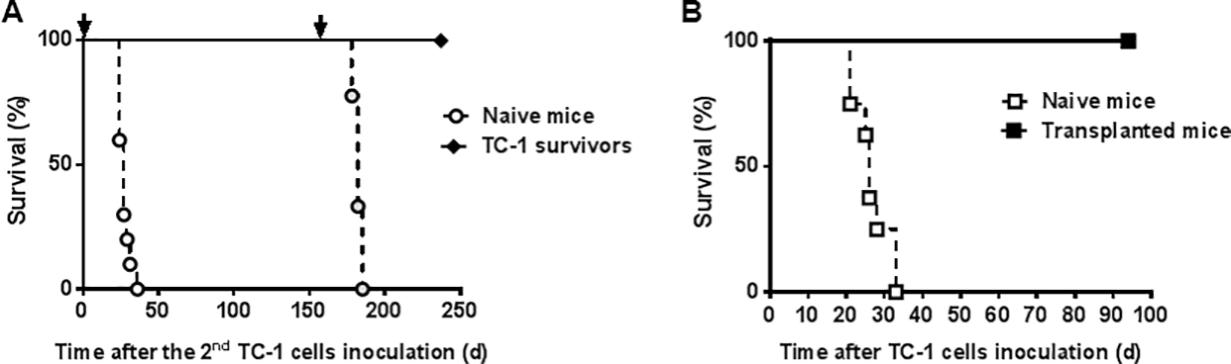
Long-term immune memory in SVP-treated TC-1 survivors. Mice treated with SVP[E7/6*] and SVP[poly(I:C)] as shown in C were (A) re-challenged at day 158 after the first TC-1 inoculation and once again 154 days later (shown by arrows) or (B) sacrificed on day 265 after initial TC-1 inoculation, and their splenocytes inoculated into naïve mice (10^8^/mouse, i.v.), which 8 days later were inoculated with TC-1 cells (8 mice/each group). No further treatment was applied.

Remarkably, delayed therapeutic treatment with the SVP vaccines enabled complete regression of tumors up to ~2000 mm^3^ in size, although generally tumors of >1000 mm^3^ were more difficult to control than those in the 500–1000 mm^3^ range (Fig 5D–5F). Individual early tumor growth curves aligned well with overall survival data with use of SVP[R848] being slightly inferior to SVP[CpG] and SVP[poly(I:C)] (Fig 5D–5F). We next re-challenged 26 surviving mice that had been treated with SVP[E7/E6*] and SVP[poly(I:C)] with TC-1 cells in the absence of any additional treatment five months after the initial tumor inoculation (and four months after the last SVP treatment). Notably none of the mice showed tumor growth for an additional five months after tumor re-challenge, at which time they were challenged with TC-1 for a third time. The treated mice remained tumor free after the third inoculation, while all control naive mice inoculated at the time of both re-challenges succumbed to tumor growth within 24-36-days (Fig 6A). Similarly, adoptive transfer of splenocytes from SVP-treated long-term TC-1 survivors led to complete protection of otherwise untreated syngeneic recipients from a challenge with TC-1 (Fig 6B), with seven of eight recipients remaining tumor-free during the 90-day observation period. The eighth animal developed a small (14 mm^3^) tumor that was subsequently eliminated (not shown).

We previously demonstrated that SVP-encapsulated R848 and CpG prevented systemic cytokine production [15]. Here, we similarly tested SVP-encapsulated poly(I:C). As expected, SVP[poly(I:C)] induced minimal levels of systemic inflammatory cytokines after subcutaneous inoculation; whereas, an identical dose of free poly(I:C) induced high levels of TNFα, IL-6, and MCP-1 (S8 Fig). The potency of both poly(I:C) forms to induce Th1 cytokines in the local draining lymph node was similar (not shown).

### Synergy of immune checkpoint inhibition or chemotherapy with SVP treatment

**Fig 7:**
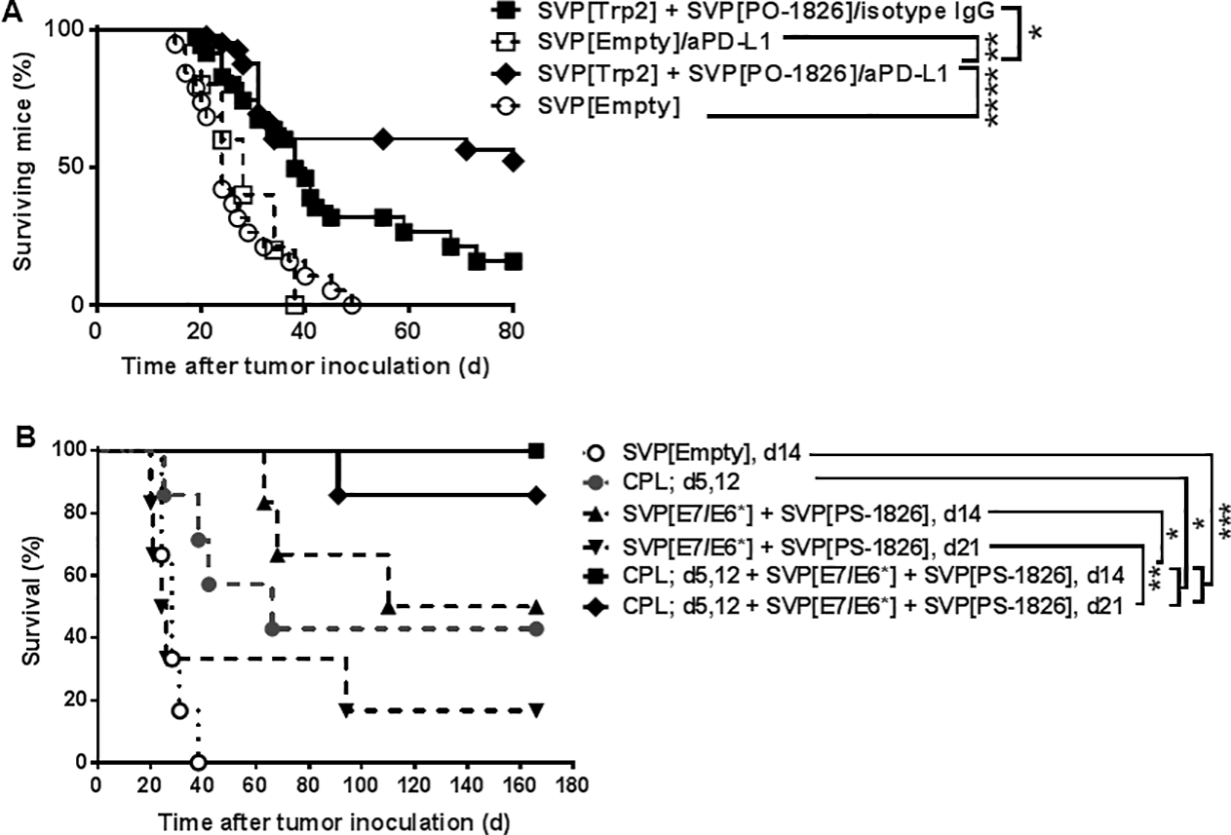
Synergy of SVP and other treatment modalities. A. SVP[Trp2.180–188] and SVP[PO-1826] were injected on d3, 7, 14 and 21 after B16-F10 tumor inoculation combined with aPD-L1 or isotype control (d6, 13, 20). Overall survival is shown; summary of 4 independent experiments. B. Synergy of cisplatin (CPL) and SVP against TC-1. CPL was administered on d5 and 12 and SVP were administered either on d14, 17, 24 and 31 (d14) or d21, 24, 31 and 38 (d21); 6–7 mice/group. * p<0.05; ** p <0.01; *** p < 0.001, **** p < 0.0001.

As demonstrated above, even suboptimal SVP immunotherapeutic treatment regimens incorporating only a single peptide epitope led to a discernible benefit against both TC-1 and B16-F10 tumors (Fig 3 and S6 Fig). These suboptimal regimens were selected to evaluate possible synergy between SVP treatment and immune checkpoint inhibitors, specifically antibodies against CTLA-4, PD-1 and PD-L1. The combination of SVP[Trp2.180–188 peptide] + SVP[PO-CpG] with anti-PD-L1 antibody showed profound synergy in the B16-F10 model (Fig 7A), leading to more than 50% long-term survival. Both anti-PD-1 and anti-CTLA-4 also demonstrated synergistic effects with SVP, but were inferior to anti-PD-L1 (not shown). None of the antibodies tested were effective without SVP (Fig 7A and not shown). Surviving mice co-treated with SVP and anti-PD-L1 or PD-1 showed superior T cell recall responses to Trp2.180–188 peptide at 12–21 weeks after the last SVP treatment compared to survivors treated with SVP alone (S9A Fig). Similarly, anti-PD-L1 antibody showed a synergistic effect against TC-1 tumor when combined with SVP[E7.I.49] + SVP[R848], while anti-PD-L1 antibody therapy alone was ineffective (S9B Fig).

SVP treatment showed even more profound synergy with chemotherapy in the TC-1 model. Cisplatin, a standard-of-care chemotherapeutic agent used in many cervical cancer treatment modalities, was partially effective as monotherapy, with ~40% overall long-term survival when administered on day 5 and 12 after tumor inoculation (Fig 7B). Similarly, SVP[E7.I.49] + SVP[CpG] monotherapy showed ~50% long term survival when treatment was initiated on d14 after tumor inoculation (Fig 7B, consistent with earlier data; see Fig 5C), but only ~17% survival when treatment was delayed until day 21. In contrast, combining cisplatin and SVP treatment regimens resulted in substantially better survival, with 100% overall survival at 166 days when SVP vaccination was initiated on day 14, and ~85% survival when SVP vaccination was delayed until day 21 after tumor inoculation.

### SVP immunogenicity in non-human primates

**Fig 8:**
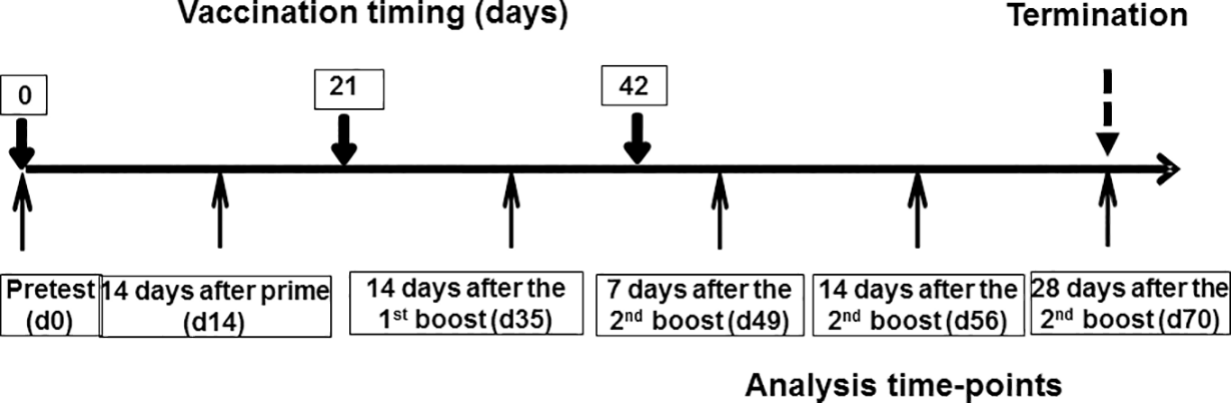
Experimental scheme for NHP immunization and sample analysis. Times of SVP vaccinations (thick arrows) are shown above the general timeline, analysis time-points (serum and PBMC isolation) are shown by thin arrows below the timeline.

A small pilot study was conducted to translate the findings in mice to nonhuman primates. In particular, since the TLR distribution differs between mice and monkeys, we were interested to compare SVP formulations of R848, PO-CpG and poly(I:C). Twelve cynomolgus monkeys (*Macaca fascicularis*) were immunized with two different dose levels of SVP-encapsulated HPV-16 E7/E6* fusion protein combined with two different dose levels of SVP[R848], SVP[PO-CpG] or SVP[poly(I:C)] (see Table 1 for details), with each animal receiving a different antigen/adjuvant dose combination (high dose E7/E6* + high dose adjuvant; high dose E7/E6* + low dose adjuvant; low dose E7/E6* + high dose adjuvant; or low dose E7/E6* + low dose adjuvant; Table 1). An additional control animal was immunized with high dose of free E7/E6* and free PS-7909. The vaccination scheme is shown in Fig 8. The various vaccine combinations were generally well tolerated. At one week after the 3^rd^ injection (day 49), nodules were observed at the immunization sites (thighs) of three animals that received the SVP[poly(I:C)] adjuvant. The animals showed no discomfort and thus no treatment was required.

**Table 1:** Non-human primate immunization with SVP and a summary of immune response readouts.

Animal	Nanoparticle Description, Dose	PBMC IFNγ ELISPOT	FACS (GRNZB/IFNγ/TNFα)	Specific CTL response	Inguinal LN IFNγ ELISPOT	IgG to E6	IgG to E7
**1**	SVP[E7/E6*] (1) + SVP[R848] (1)	**–**	**+/–/–**	**–**	**–**	**++**	**+++**
**2**	SVP[E7/E6*] (1) + SVP[R848] (2)	**+**	**–/–/+**	**Not done**	**Not done**	**+**	**++**
**3**	SVP[E7/E6*] (2) + SVP[R848] (1)	**+/–**	**–/+/–**	**–**	**–**	**++**	**++**
**4**	SVP[E7/E6*] (2) + SVP[R848] (2)	**–**	**–/–/–**	**+**	**–**	**+**	**++**
**5**	SVP[E7/E6*] (1) + SVP[PO-7909] (1)	**++**	**+/+/–**	**+**	**–**	**++**	**++**
**6**	SVP[E7/E6*] (1) + SVP[PO-7909] (2)	**–**	**–/–/–**	**Not done**	**+/–**	**+**	**++**
**7**	SVP[E7/E6*] (2) + SVP[PO-7909] (1)	**++**	**+/+/–**	**Not done**	**+/–**	**++**	**++**
**8**	SVP[E7/E6*] (2) + SVP[PO-7909] (2)	**–**	**–/–/–**	**–**	**–**	**+/–**	**+**
**9**	SVP[E7/E6*] (1) + SVP[poly(I:C)] (1)	**++**	**+/+/+**	**+**	**+/–**	**++++**	**++++**
**10**	SVP[E7/E6*] (1) + SVP[poly(I:C)] (2)	**++**	**+/+/+**	**–**	**+**	**+++**	**+++**
**11**	SVP[E7/E6*] (2) + SVP[poly(I:C)] (1)	**+**	**+/+/–**	**+**	**–**	**+++**	**+++**
**12**	SVP[E7/E6*] (2) + SVP[poly(I:C)] (2)	**++**	**+/+/+**	**+**	**+**	**++**	**++**
**13**	E7/E6* (1) + PS-7909 (1), free	**–**	**–/–/+**	**Not done**	**–**	**++**	**++**

**Dosage:** E7/E6* (1) and (2)– 200 μg and 50 μg; R848 (1) and (2)– 200 μg and 40 μg; human-specific CpG ODN PO-7909 (1) and (2)– 600 μg and 200 μg; poly(I:C) (1) and (2)– 1500 μg and 300 μg. **ELISPOT grading**: ‘++’ is >10-fold over the background; ‘+’ is 3-10-fold over the background; ‘+/-’ is 2–3 fold over the background; ‘–’ is <2-fold over the background. **ICS and CTL grading:** ‘-’–no elevation over the background at any time-point, ‘+’–clear elevation over the background and also over day 0 levels. **ELISA grading.** E6: ‘+/-’ 2-3-fold over the background; ‘+’ EC50 >3-fold over the background, but <3,000 at all points, ‘++’ EC50 >5,000 at least at one time-point, ‘+++’ EC50 >10,000 at multiple points and ‘++++’ EC50 >20,000 at multiple points. E7: ‘+’ EC50 <10,000, ‘++’ EC50 >10,000, ‘+++’ EC50 >50,000 and ‘++++’ EC50 >100,000 at multiple points.

**Fig 9:**
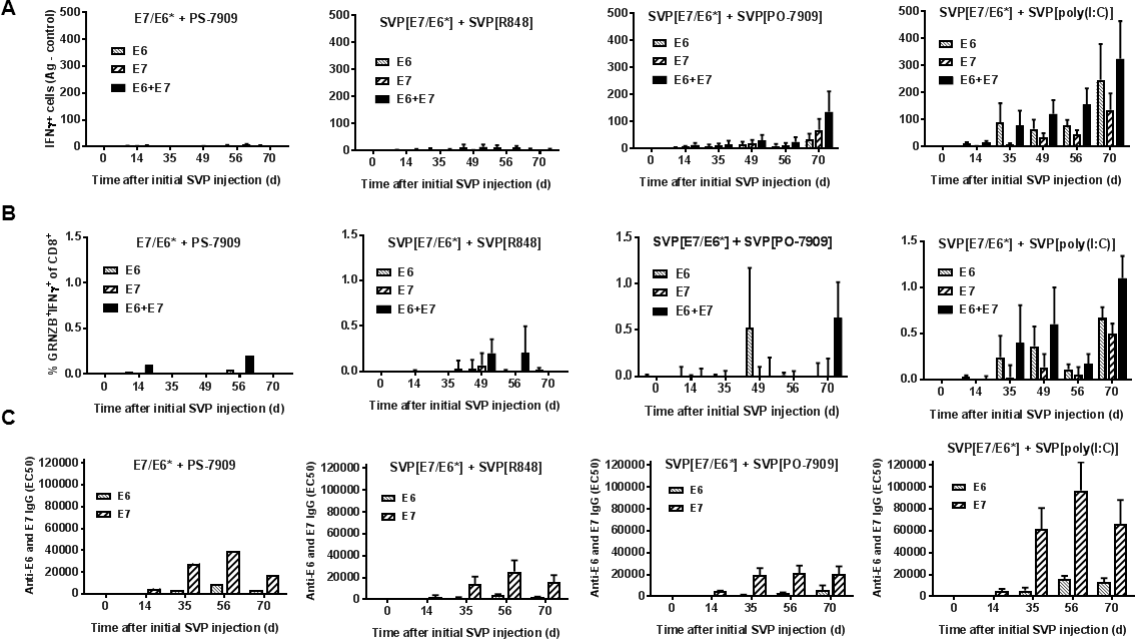
Induction of T and B cell responses to HPV-16 oncogenic proteins by SVP in non-human primates. A. IFNγ ELISPOT in PBMC. B. IFNγ^+^Grnzb^+^ share of CD8^+^ cells in PBMC after peptide stimulation followed by FACS analysis. Peptide pools used for PBMC stimulation are indicated. C. Induction of antibodies to HPV-16 E6 and E7 proteins. Animals in A-C are grouped according to an adjuvant used with every group containing 4 animals immunized either with high or low doses of E7/E6* (2 each, 200 and 50 μg).

Animals were assessed for antigen-specific T cell recall by interferon-γ (IFN-γ) ELISPOT, CD8^+^ T cell expression of granzyme B, IFN-γ, and TNF-α, CTL activity against E6 and E7 and anti-E6 and anti-E7 antibody responses (see summaries for IFN-γ ELISPOT, granzyme B and IFN-γ intracellular staining and humoral response to E6 and E7 in Fig 9, data for individual animals is shown in S10 and S11 Figs; results for each readout in individual animals are summarized in Table 1). Monkeys immunized with SVP[E7/E6*] adjuvanted with SVP[poly(I:C)] showed the most robust T cell recall responses, which were detectable as early as day 35 and increased over time (Fig 9A and 9B, and S10 and S11 Figs). SVP]R848] and SVP[PO-CpG] induced weaker T cell recall responses, while the control animal treated with free PS-CpG showed no detectable response. All four animals treated with SVP[E7/E6*] adjuvanted with SVP[poly(I:C)] showed CD8 T cells positive for granzyme B and IFN-γ expression and three of the four animals demonstrated HPV E6/E7-specific CTL activity (Table 1). Moreover, SVP[poly(I:C)] adjuvant induced superior B cell responses as well, with antibody levels to E6 and E7 proteins being 3–4 times higher than in animals treated with SVP]R848] or SVP[PO-CpG] (Fig 9C, Table 1). These results indicate that the SVP[poly(I:C)] is the superior adjuvant in NHP for the induction of antigen-specific CD8 T cell and B cell responses.

## Discussion

Nanoparticles have been shown to be effective vehicles for vaccine delivery, as they are capable of trafficking via the draining lymphatics to lymph nodes where they are selectively taken up by antigen presenting cells ([45], reviewed in [46, 47]). Here we describe the development of a biodegradable polymer-based nanoparticle formulation for efficient induction of CTL responses. Our objectives were to optimize therapeutic vaccines by assessing different polymer formulations, TLR ligands, tumor antigens, and synergy with checkpoint inhibitors and chemotherapeutic agents. Finally, we have translated key findings in a small pilot study in nonhuman primates.

SVP are comprised of biodegradable polymers. We assessed SVP made with poly(lactic-co-glycolide) (PLGA) and poly(lactic acid) (PLA), which are predicted to have different properties with respect to their degradation time in vivo. SVP vaccines comprised of PLA as a core polymer induced potent CTL responses in vivo with kinetics that are typical of most vaccines, namely with CTL activity peaking at day 7 and then rapidly contracting. In contrast, SVP vaccines comprised of PLGA as a core polymer induced durable CTL activity that was sustained for up to several weeks (Figs 1A–1E, 2B and 5A and S1 Fig). The uptake of fluorescent-labeled SVP-PLGA and SVP-PLA particles by antigen-presenting cells in the draining lymph node was similar (S3 Fig). However the use of DQ-OVA revealed that the SVP-PLGA formulation enabled more extensive and sustained proteolysis of the antigen (S3 Fig), resulting in an increase in activated effector CD8 T cells (Fig 1). This is likely due to the faster degradation rate of PLGA compared to PLA under acidic conditions such as those found in the endosomes of antigen presenting cells. The enhanced processing of antigen in the SVP-PLGA formulation may thus be related to the ability of professional antigen presenting cells to more completely degrade the polymer matrix and encapsulated antigen within SVP-PLGA compared to SVP-PLA. In addition, the PLGA polymer core provides higher antigen loads, likely producing a denser protein corona on the nanoparticle surface, which would be more readily accessible to endosomal protein processing machinery. Collectively, it is likely that PLGA formulations improve cellular immune responses by promoting prolonged processing and presentation of the encapsulated antigen.

TLR ligands have potent adjuvant properties by activating innate antigen presenting cells and promoting the production of Th1 cytokines. One of the concerns of using TLR agonists as adjuvants is the production of systemic inflammatory cytokines. We focused here on TLR3, TLR7/8, and TLR 9 agonists, as these receptors reside within the endosomal compartment. When SVP are endocytosed, they can release their payload directly to adjacent receptors. We have previously shown that multiple immunizations with SVP[R848] and SVP[CpG] formulations initiate local, but not systemic, cytokine induction [15] and induce minimal inflammation at the site of vaccine administration compared to free TLR agonists. We extend these findings here to SVP[poly(I:C)], a TLR 3 agonist (S8 Fig). All three formulations were effective adjuvants for promoting potent CTL responses in mice (see below for discussion of relative activity in nonhuman primates), although SVP[CpG] and SVP[poly(I:C)] induced more sustained effector CD8 T cell activity (Fig 5A). Importantly the SVP-formulated adjuvants supported potent therapeutic vaccine efficacy in tumor models. Even mice with palpable tumors up to 2000 mm^3^ in size showed substantial tumor regression and long term survival with therapeutic SVP vaccination (Fig 5D–5F).

Both peptide antigens and protein antigens can be encapsulated in SVP vaccines. We have previously demonstrated that a MHC class II-binding peptides can be encapsulated to provide T cell help to B cells [48]. Here we focused on the ability of SVP vaccines encapsulating MHC class I-binding peptides to induce CD8 T cell responses. An SVP vaccine encapsulating the Trp2.180–188 peptide adjuvanted with SVP[PO-CpG] or SVP[R848] provided therapeutic protection in the B16-F10 melanoma model (Fig 6A and S6C Fig). Similarly, SVP[E7.I.49] encapsulating the dominant MHC class I epitope of the HPV E7 antigen, provided significant benefit when dosed therapeutically in the TC-1 tumor model. A major concern of using peptide vaccines is the incomplete coverage of potential epitopes and the MHC heterogeneity in the human population. However, protein vaccines are often limited by inefficient cross-presentation of endocytosed antigens. SVP vaccines containing E7* protein or E7/E6* fusion protein induced potent CD8 T responses to both major MHC class I epitopes as well as subdominant epitopes (Fig 4A and 4B). In addition, SVP[E7*] and SVP[E7/E6*] vaccines provided superior therapeutic activity compared to the SVP[E7.I.49] peptide vaccine (Fig 4C). In particular, SVP[E7/E6*] adjuvanted with SVP[poly(I:C)] provided ~80% survival even when treatment was initiated 10 days after tumor inoculation and more than 50% survival when treatment was delayed until day 14, at which time mice had palpable tumors of approximately 500 mm^3^ (Fig 5B, 5C and 5F). It is noteworthy that the SVP injections were administered into tumor-distal sites (hind limbs), not peritumorally. Our results compare favorably to several other experimental HPV therapeutic vaccine candidates tested in the TC-1 tumor model, which typically used more aggressive treatment regimens [49–54].

Tumors can create an immunosuppressive microenvironment, and render the tumor refractory to treatment [9, 10]. The recent approval of immune checkpoint inhibitors have researchers pursuing combination therapy strategies. We show here that SVP vaccines are synergistic with checkpoint inhibitors, such as anti-PD-L1 antibodies (Fig 6A and S9B Fig). A suboptimal single-peptide SVP vaccine showed significant synergy with anti-PD-L1 antibodies, leading to long term survival of greater than 50% mice with durable immune memory in the aggressive B16-F10 melanoma tumor model. Even more striking therapeutic efficacy was observed when the SVP therapy was combined with cisplatin, a chemotherapeutic agent that is often used to treat cervical cancers. Cisplatin alone was only partially effective in the TC-1 model, in agreement with other studies [55, 56]. However, cisplatin was combined with SVP therapy provided 100% long term survival even with SVP treatment was delayed until 13/14 days after tumor inoculation and >80% survival when SVP treatment was delayed until 21 days (Fig 6B). Re-inoculation of SVP[E7/E6*]-immunized survivors with TC-1 tumor cells led to complete tumor rejection reflecting durable immune memory to E7 antigen (Fig 5G and 5H).

One of the hurdles to cancer vaccine development is that data in mice have not translated to humans [9]. We conducted a small pilot study to assess the immunogenicity of SVP[E7/E6*] in non-human primates. In particular, we focused on evaluating different TLR agonists, as TLR expression differs between mice and humans [57] and the subset of dendritic cells implicated in cross presentation is also different [57]. We evaluated three SVP-encapsulated TLR agonists, R848, PO-CpG and poly(I:C). While antigen-specific T cell activity and antibody production was seen in animals from all experimental arms, formulations adjuvanted with SVP[poly(I:C)], a TLR3 agonist, generated the most robust responses. Notably, CD8 T cells from all four animals treated with SVP[E7/E6*] adjuvanted with SVP[poly(I:C)] were positive for granzyme B and interferon-γ expression and three of the four animals demonstrated HPV E6/E7-specific CTL activity (Table 1). These results indicate that the SVP[poly(I:C)] is the superior adjuvant in NHP for the induction of antigen-specific CD8 T cells. These results are consistent with TLR3, but not TLR 7, 8, or 9, being highly expressed on the subpopulation of human dendritic cells capable of efficient antigen cross-presentation [57].

Our data indicate the therapeutic potential of an SVP-based HPV vaccine. Notably, SVP technology shows the potential to augment the immunogenicity of the target antigen via its particulate delivery and sustained in vivo release, resulting in robust and sustained production of effector CD8 T cell activity with durable immunological memory. In addition SVP adjuvants can deliver potent TLR agonists, such as poly(I:C) to their cognate receptor in the endosome, thereby reducing the potential for systemic side-effects. Moreover, GMP manufacturing of SVP nanoparticles has been also shown to be scalable and reproducible [58]. Therefore a therapeutic SVP platform-based vaccine against HPV-induced malignancies is an attractive candidate for further clinical evaluation.

## Supporting information

S1 Methods Supporting information materials and methods. (DOCX)

S1 Fig Long-term induction of antigen-specific cytotoxicity by SVP-PLGA. Animals (3–6 per time-point) were injected with SVP[OVA]-PLGA combined with SVP[R848] and CTL activity measured in vivo at times indicated (percentage of cells killed is shown for each time-point).
(DOCX)

S2 Fig PCR amplification of OVA-specific DNA sequences from EG.7-OVA tumors from SVP-treated mice. N1, N2 –irrelevant TC- 1 tumor tissues. Samples 1–3, 6, 7 and 11 –mice treated with SVP[Empty], tumor tissues taken at sacrifice on days 18 to 21; samples 4, 5, 8–10 and 12 –mice treated with SVP[OVA]-PLGA and SVP[R848], tumor tissues taken at sacrifice on days 24 to 32. **A**, **B**–amplification with two different primer pairs located within OVA gene (expected sizes 814 and 424 bp, correspondingly), **C**– β-globin-specific primers, M– 100 bp ladder MW markers (500-bp and 1000-bp fragments indicated).
(DOCX)

S3 Fig Local SVP trafficking and antigen processing. Mice were injected with SVP[OVA]-PLA or SVP[OVA]-PLGA containing Cy5-labeled polymer and DQ-labeled OVA. At 2–48 hours draining LN were taken, processed and stained with surface marker antibodies and analyzed by FACS. Shares of cell populations emitting Cy5- and DQ-based fluorescence (top and bottom rows, correspondingly) are shown for B cells (**A**) and different DC subtypes (**B-E**).
(DOCX)

S4 Fig SVP induce long-term immune memory. **A, B.** Mice were immunized at d0, 21 and 42 with SVP[OVA] + SVP[R848] (**A**) or with SVP[OVA] + SVP[CpG] with free and SVP-entrapped PS- and PO-forms of CpG ODN 7909 (**B**). Anti-OVA IgG titers were measured at d122 and d721 (**A**) or at d33 and d372 (**B**). **C.** Late boost of pre-immunized mice. Mice described in **A** were split into three groups and boosted at d742 with SVP[R848] + SVP[OVA] at different doses of OVA (shown at X-axis) and titers measured on d721, 754, and 768. **D, E.** Antigen-specific IFN-γ induction in PBMC from long-term immunized animals. **D.** Mice were immunized with SVP[R848] + SVP[OVA]-PLGA; direct ex vivo ELISPOT with OP.I.257 peptide was run within 200–400 days after the last SVP immunization. **E.** Mice were immunized (3 times; d0, 21, 42)) with SVP[OVA]-PLGA combined with SVP-entrapped PS-/PO-CpG or free PS-CpG; direct ex vivo ELISPOT was run at 330d after the last SVP immunization.
(DOCX)

S5 Fig Decrease of local inflammation after injections of SVP[PO-CpG]. SVP-entrapped PO- or free CpG PS-1826 injections were carried out on days 0, 4, 11 and 18 (**A**) or days 0, 21 and 42 (**B**). Footpad thickness was measured daily. Means with SD are shown.
(DOCX)

S6 Fig Anti-tumor activity of SVP. **A, B.** Inhibition of TC-1 lung seeding by SVP treatment. TC-1 cells were injected i.v., mice were treated at d3, 7, 14 and 21 and lungs harvested on d32. Metastatic counts (**A**) and lung appearance (**B**) for SVP- and mock-treated groups are shown with arrows pointing at typical pronounced metastases in lungs from all the untreated mice. **C.** Treatment of B16-F10 tumors by SVP[Trp2] combined with R848 or CpG ODN PO-2395. Survival proportions are shown. Treatments administered on days 3, 7, 14 and 21 (indicated by arrows; 10–15 mice/group). **–p < 0.01, *** p<0.001, **** p < 0.0001.
(DOCX)

S7 Fig Immunogenicity of SVP-entrapped HPV-16 antigens. Induction of E7-specific antibodies by SVP[E7*] and SVP[E7*E6*] (co-injected with SVP[R848]; 5 mice/group). * p <0.05; **p<0.01.
(DOCX)

S8 Fig Systemic cytokine induction after subcutaneous inoculation of free, but not SVP-encapsulated TLR3 agonist poly(I:C). Mice were inoculated with either SVP[E7/E6*], SVP[E7/E6*] combined with SVP[poly(I:C)] or admixed with free poly(I:C) and bled at times indicated. The same amount of free or SVP-encapsulated poly(I:C) was used for each group (5 μg). Serum samples were collected at the times indicated and analyzed for individual cytokines by ELISA. Average cytokine concentration is shown (three samples per group per each time-point). **A**–TNF-α, **B**–IL-6, **C**–MCP-1.
(DOCX)

S9 Fig Synergy of SVP and immune checkpoint inhibitors leads to higher survival and immune memory. **A.** Immune memory in SVP-treated B16 survivors from experiments described in **Fig 5I**. Trp2-specific IFN-γ production in splenocytes of surviving mice (taken on days 104–171 after initial inoculation or days 83–150 after the final SVP treatment) was measured after overnight stimulation. Numbers of mice in each group is shown in parentheses. **B.** SVP[E7.I.49] and SVP[R848] were injected on days 14, 17, 24 and 31 after TC-1 inoculation either alone or combined with antibody to PD-L1 or isotype control administered on days 18, 21 and 25 (7–8 mice/group). Overall survival is shown (* p<0.05; ** p <0.01).
(DOCX)

S10 Fig IFNγ ELISPOT in monkey PBMC; individual graphs. Adjuvant and E7/E6* doses specified per Table 1 (high dose– 1, low dose– 2), data grouped in columns per adjuvant used (indicated on top of each set); peptide pools used for PBMC stimulation are shown. Y-axis scale for R848 is 8 times smaller than for CpG and poly(I:C) with the latter two being equal (with the exception of low doses of both E7/E6* and an adjuvant).
(DOCX)

S11 Fig Granzyme B^+^IFNγ^+^ fractions from total CD8^+^ monkey PBMC after peptide stimulation; individual graphs. Adjuvant and E7/E6* doses specified per Table 1 (high dose– 1, low dose– 2), data grouped in columns per adjuvant used (indicated on top of each set); peptide pools used are shown. Y-axis scale for all adjuvants is identical with the exception of two graphs for both CpG (high adjuvant, low and high E7/E6*) and poly(I:C) (high adjuvant, low E7/E6* and high E7/E6*, low adjuvant), which are of larger scale.
(DOCX)
