# Supplementary material for: Synthetic vaccine particles for durable cytolytic T lymphocyte responses and anti-tumor immunotherapy
Source: PLoS One. 2018 Jun 1;13(6):e0197694. doi: 10.1371/journal.pone.0197694 (PMC5983463; doi:10.1371/journal.pone.0197694)
Supplement: S1 Methods — (DOCX) [file pone.0197694.s001.docx]

**Materials and Methods**

**Cells and reagents**

Cell lines E.G7-OVA (mouse thymoma transfected with OVA), melanoma B16-F10 and TC-1, expressing HPV-16 E6 and E7 oncoproteins [44] were purchased from the ATCC (American Type Culture Collection, Rockville, MD, USA) and grown per manufacturer’s recommendations. HPV-16 E7 peptide sequence RAHYNIVTF and TRP2.180-188 peptide sequence SVYDFFVWL were purchased from Peptides International (Louisville, KY USA). Subdominant HPV-16 E7 peptides (DTPTLHEYM; STHVDIRTL and FCCKCDSTL) were purchased from IBMH EkoBioFarm Ltd (Moscow, Russia). HPV-16 E7 and E7/E6 fusion recombinant proteins (full size E7 with C24G and E26G mutations abolishing Rb binding and the same sequence followed by aa 9-121 of E6 with F54R and L57G mutations abolishing p53 binding and increasing E6 solubility [Supporting Refs. 1-3]) were synthesized by A.V. Kozyr (Applied Microbiology Center, Moscow, Russia). Ovalbumin was purchased from Worthington Biochemical Corporation (Lakewood New Jersey, USA). DQ™ Ovalbumin (DQ-OVA) was purchased from Thermo Fisher Scientific (Waltham, MA USA). CpG-containing oligonucleotide (CpG) 7909 and 2395 in phosphorothioate (PS) and phosphodiester (PO) forms were purchased from Nitto Denko Avecia (Milford, MA USA). CpG PO-1826 and PS-1826 were purchased from Oligo Factory (Holliston, MA USA). Poly(I:C) was purchased from InvivoGen (San Diego, CA USA).

**Chemicals**

PLGA-Cy5 or PLA-Cy5 fluorescent polymer conjugates were prepared at Selecta, using carbodiimide chemistry by the reaction of N-hydroxysuccinimidyl (NHS) activated PLGA or PLA with acid reactive Cy5 dye, respectively, in dichloromethane followed by repeated washing and precipitation of the resulting conjugates to remove unreacted free Cy5 and drying under vacuum. Poly(lactide) or poly(lactide-co-glycolide) polymers (PLA and PLGA respectively), and their poly(ethylene glycol), (PEG), block copolymers (PLA-PEG), were purchased from Evonik Industries (Essen, Germany), unless otherwise specified. Resiquimod-polymer prodrug (PLGA-R848), was custom synthesized by Princeton Global Synthesis (Bristol, Pennsylvania USA).

**Nanoparticle preparation**

PLGA-R848 nanoparticles were prepared using the single emulsion method by dissolving PLA-PEG, PLGA-R848, and PLA, at a ratio of 1:2:1 respectively, in CH_2_Cl_2_ at 10% wt/vol. Polymer organic phase was added to a thick walled glass pressure tube with an aqueous solution of PVA at 65 mg/mL in PBS at a ratio of 1:3 (v/v), and vortex mixed. Emulsification was carried out using a Branson Digital Sonifier model 250 equipped with a 1/8” tapered tip sonic horn with a model 102C converter, from Branson Ultrasonics (Danbury, Connecticut USA). The mixture was emulsified at 30% amplitude for 1 minute. The nanoemulsion was then poured into a beaker with PBS and allowed to evaporate for 2 hours under stirring. The hardened nanoparticles were washed twice in PBS, sterile filtered, and stored at -20°C.

For the double emulsion formulations, the antigens and adjuvant were dissolved into aqueous solutions as described [15]. OVA was dissolved at 50 mg/mL in PBS and DQ-OVA was prepared by mixing with OVA at a 1:4 w/w ratio in PBS, at a total concentration of 20 mg/mL protein. CpG and poly(I:C) solutions were prepared as described [Supporting Ref. 4]. HPV 16 E7 peptide was prepared at 10 mg/mL in 0.13M HCl and 10% formamide v/v. TRP2 peptide was prepared in 50mM NaOH and 20% sucrose w/v. Recombinant HPCV-16 proteins were dissolved in 20 mM TRIS-HCl pH 7.5, 300 mM NaCl, 2 mM DTT, 1 mM PMSF. To form the polymer organic phase, PLGA (or PLA), and PLA-PEG were co-dissolved at a ratio of 3:1 to a combined 10% w/v polymer in CH_2_Cl_2_. To form the inner W/O emulsion, the antigen or adjuvant aqueous phase was added to a thick walled glass pressure tube with the polymer organic phase at a ratio of 1:5 v/v, and homogenized via sonication at 50% amplitude for 40 seconds. To form the W/O/W double emulsion, an aqueous solution of PVA in phosphate buffer was added at a ratio of 1:3 v/v to the organic phase, vortex mixed, and homogenized via sonication at 30% amplitude for 60 seconds. The double emulsion was then dispersed into PBS and processed the same as the single emulsion.

The SVP appear as monodisperse, spherical nanoparticles by transmission electron microscopy (TEM), or vitrified specimens by cryo-TEM. The average load of adjuvant-containing SVP was in 3.4-5.2% range. The loads of antigen-containing SVP were within 0.9-1.3% for peptides, 4-4.5% for OVA, 1.5% for E7* and 2.0% for E7/E6*. By dynamic light scattering, SVP had a size range of 99-188 nm, with polydispersity index of 0.005-0.121. We have not observed aggregation of these formulations under ordinary use which includes production, freeze-thaw cycles, or storage at -20°C. Surface charge was not measured.

**Flow cytometry**

Cells were stained pairwise with antibodies against the following mouse surface cell molecules: CD8α, CD19, CD62L, CD44, B220 and CD11c (BD Biosciences, CA, USA). The gating logic was as follows: plasmacytoid dendritic cells (CD11c+, B220+), myeloid dendritic cells (CD11c+, B220−), conventional dendritic cells (CD11c+CD8α+), pan-DC (CD11c+) and B cells (CD11c−, B220+). Similarly, SIINFEKL-loaded pentamers (Proimmune, Oxford, UK), were used along with anti-mouse CD8α and CD19 (to gate out non-specific pentamer binding) and also stained with anti-CD62L and CD44 with central memory and effector memory populations defined as CD62L^high^CD44^high^ and CD62L^low^CD44^high^), respectively. Cell samples were then washed and immediately analyzed by flow cytometry. Data were analyzed with FlowJo software (Tree Star Inc., Ashland, OR, USA).

**Analysis of tumor-infiltrating lymphocytes**

Mice were inoculated with TC-1 cells and then treated with SVP[E7/E6*] combined with SVP[Poly(I:C)] or SVP[Empty] on days 14, 17, 24 and 31. Tumors were taken from SVP[Empty]-treated animal on d31 and from therapeutic SVP-treated animal on d59 and harvested into ice-cold RPMI, cut and minced into 1-2 mm pieces with a sterile scalpel in 100 mm Petri dish. Tissues were then transferred into a 50-ml tube, centrifuged, resuspended in 10 ml of RPMI, collagenase type IV added (200 U/ml) and incubated at 37°C for 30 min. The resulting cell suspensions were filtered through a sterile nylon mesh, spun down, resuspended, filtered and stained for surface CD3 molecule with of anti-mouse CD3-FITC antibody (e-Bioscience, San Diego, CA) followed by flow cytometry and data analysis (BD, FACSDiva^TM^ v7.0 software, San Jose, CA).

**PCR analysis of tumor tissues**

Tumors were taken at sacrifice and kept at -80⁰C prior to analysis, then minced, homogenized (100-200 mg of sample solubilized overnight at 56⁰C in 1 ml of ATL tissue lysis buffer in the presence of proteinase K) and genomic DNA isolated using DNeasy blood and tissue kit (Qiagen, Venlo, Netherlands) from 100-200 µl of the solubilized sample according to the manufacturers manual. Samples were then quantitated on Nano-drop (Thermo Fisher), uniformly diluted to 50 ng/µl, run on 1% agarose gel for confirmation upon which 100 ng of genomic DNA used in 20 µl PCR reaction with HotStarTaq DNA-polymerase. (Qiagen) and either OVA-specific (forward 5’-GAAGAGAGATACCCAATCCTGC-3’ and reverse 5’-CTTCATCAGGCAACAGCACCAAC-3’ or 5’-AGAAGAGAACGGCGTTGGTTGC-3’) or β-globin control primers in standard conditions (dNTP at 200 µM, each primer at 0.3 µM; 15 min, 95⁰C denaturation/enzyme activation) followed by 30 cycles of 45 sec at 94⁰C, 2 min at 60⁰C and 2 min at 72⁰C, concluded with 7 min at 72⁰C) and resulting products visualized on 1% agarose gel (6 µl per lane).

**Enzyme-linked immunospot assay** (**ELISPOT)**

ELISPOT plates (MultiScreen-IP, Millipore) were coated with purified anti-IFN-γ monoclonal antibody (clone AN18, Mabtech). Isolated PMBC were cultured via limiting dilution in the presence or absence of TRP2 at 1 µg/mL for 18h at 37°C. Positive and negative controls included Concanavalin (10 µg/mL) and medium alone respectively. Plates were developed by subsequent incubations with biotinylated anti-IFN-γ antibody, then Streptavadin-HRP (clone R4-6A2-biotin, Mabtech). Plates were analyzed via automated ELISA-spot automated plate reader (Zellnet Consulting, Fort Lee, NJ). The average of duplicate counts of IFN-γ spot forming cells (SFC) was calculated subtracting background spots (if any) from antigen specific for each cell concentration.

### **Determination of cytokine concentration in sera and culture supernatants**

Local cytokine secretion was determined in culture supernatants after in vitro incubation of draining lymph nodes (LNs) from immunized animals. Briefly, popliteal LN were harvested at different intervals after injection of SVP, free TLR agonist, or PBS and incubated individually without processing for 16 h in 300 μl of complete RPMI 1640. Culture supernatants were then assayed for murine cytokines by CBA array kit (BD Biosciences, CA, USA). Cytokine levels determined in the cultures from LNs of PBS-immunized animals were used as the initial time-point (0 h). Systemic cytokine levels in individual serum samples drawn at different time-points after vaccination were also measured by CBA array kit with levels from the sera of control animals considered as initial time-point (0 h).

### **Determination of antibody titers by ELISA**

96-Well Costar plates (Corning Inc., Corning, NY, USA) were coated with 100 μl per well of OVA or HPV16 E7* protein (5 μg/mL) and incubated overnight at 4 °C. Plates were washed three times with 0.05% Tween-20 in PBS, then 300 μL of diluent (1% casein in PBS; Thermo Fisher, Waltham, MA, USA) was added to each well to block non-specific binding, and plates were incubated for 2 h at room temperature (RT). Plates were washed as described above, and serum samples were serially diluted 3-fold down the plate and incubated for 2h at RT. Naive mouse serum was used as a negative control. Plates were washed and horseradish peroxidase (HRP)-conjugated goat anti-mouse IgG (Abcam) detection antibodies was added to each well. Plates were incubated in the dark for 1h at RT and washed (three times, with at least a 30-s soak between each wash). TMB substrate (BD Biosciences, San Jose, CA, USA) was added, and plates were incubated for 25 min in the dark. The reaction was stopped by adding stop solution (2N H_2_SO_4_) to each well, and the OD was measured at 450 nm with subtraction of the 570 nm reading using a Versamax plate reader (Molecular Devices, Sunnyvale, CA, USA). Data analysis was performed using SoftMax Pro v5.4 (Molecular Devices).

**NHP Studies**

**ELISPOT (PBMC)**

ELISPOT assays were performed using human IFN-γ kit (CTL, Cleveland, OH) according to the manufacturer’s protocol. A pre-coated Elispot plate was washed with DPBS once and then 100 µL of CTL test medium supplemented with 2 mM L-Glutamine or antigen/mitogen solution was added and incubated in a 37ºC humidified incubator supplied with 5% CO_2_ for 15-30 minutes. 3×10^5^ PBMC in 100 µL of CTL test medium were then added in triplicate wells and the plates were incubated for 24 hours in a 37ºC humidified incubator supplied with 5% CO_2_. 10^5^ PBMC were stimulated with PHA at 2 µg/mL. HPV peptide pools were purchased from JPT Peptide Technologies GmbH (Berlin, Germany) and used at a final concentration of 1 µg/mL for each peptides in the pool. Plates were washed two times with DPBS and two times with DPBS containing 0.05% Tween 20. 80 µL of human IFN-γ detection antibody solution (1:250) was then added to each well and plates incubated at room temperature for two hours, then washed three times with DPBS containing 0.05% Tween 20, then 80 µL of diluted streptavidin-AP conjugate solution was added and plates incubated for 30 minutes at RT. Plates were then washed two times with DPBS containing 0.05% Tween 20 and two times with distilled water, then 80 µL of developer solution was added to each well and plates were developed in the dark for 15 minutes. The protective underdrain of the plates was removed and the reaction was stopped by washing the plates in running distilled water. Plates were dried overnight and scanned/counted using a CTL-ImmunoSpot S6 Macro Analyzer (CTL, Cleveland, OH).

**ELISPOT (lymph nodes)**

Antigen specific T cell recall from axillary, inguinal, and mesenteric lymph nodes was measured. ELISPOT plates (MultiScreen-IP, Millipore) were coated with purified anti-IFN-γ monoclonal antibody (clone MT126L, Mabtech). PMBC were cultured via limiting dilution in the presence or absence of E6 peptide, E7 peptide, or E6/E7 peptides (pm-hpv16-E6, pm-hpv16-E7, JPT Technologies) at 1 µg/mL for 18h at 37°C. Positive and negative controls included Concanavalin (10 µg/mL) and medium alone respectively. Plates were developed by subsequent incubations with biotinylated anti-IFN-γ antibody, then Streptavadin-HRP (clone 7B6-1-biotin, Mabtech). Plates were analyzed via automated ELISA-spot automated plate reader (Zellnet Consulting, Fort Lee, NJ). The average of duplicate counts of IFN-γ spot forming cells (SFC) was calculated subtracting background spots (if any) from antigen specific for each cell concentration.

## **In vitro PBMC Stimulation for FACS Analysis**

100 µL of PBMC resuspended at 1x10^7^ cells/mL was mixed with 100 µL of peptide solutions. These included actin peptide (1 µg/mL) negative control, E6 peptide pool (1 µg/mL), E7 peptide pool (1 µg/mL) and E6 + E7 peptide pools (2 µg/mL) with final concentration of 1 µg/mL each of the peptides in the pools, and also PMA (50 ng/mL) + ionomycin (1 µg/mL) controls. Cells were cultured in a final volume of 200 µL for a total of 20 hours in a 5% carbon dioxide (CO_2_) incubator at 37°C, except for PMA/Ion, which was not added until the last 4 hours. For the degranulation assay, anti-CD107a antibody or isotype control was included during the last 4 hours of incubation. Monensin was added during the last 4 hours of incubation to inhibit cytokine secretion.

## **Surface and Intracellular Staining and Flow Cytometry Analysis**

PBMC were washed and incubated with fixable viability dye (FVD), followed by staining with antibodies to surface antigens, including CD3-FITC or AF700, CD4-PerCP/Cy5.5, CD8-V500, CD14-APC/Cy7, CD20-APC/Cy7, CD28-BV421, CD45-AF700 and CD95-APC. Thereafter, cells were washed, fixed, and permeabilized for intracellular staining with antibodies to granzyme B-PE-CF594, IL2-PE/Cy7, TNFα-PE, and IFNγ-PE/Dazzle594. All antibodies were anti-human from BD Bioscience (San Jose, CA) or BioLegend (San Diego, CA). Samples were analyzed using flow cytometry in two 10-color panels of antibody staining for degranulation and cytokine assays.

Gallios Flow Cytometer (Beckman Coulter Inc., Brea, CA) was used to acquire events for data analysis. Data analysis was performed off-line using the Kaluza (Beckman Coulter) Flow Cytometry Analysis Software (version 1.2). The PBMCs were gated using side-light scatter (SS) and forward‑light scatter (FC). A dot plot of FS PEAK versus FS area (FS INT) was used to discriminate cell doublets/aggregates. Fixable viability dye (FVD) was included to exclude dead cells from data analysis. Viable CD3^+^ T lymphocytes gated on FVD^-^, CD3^+^, CD45^+^, CD14^-^, and CD20^-^ populations were acquired for 50,000 events, where possible. The acquired populations were then assessed for the expression of CD107a, granzyme B, IFNγ, TNFα, and IL-2 by cytotoxic T cells (CD3^+^CD8^+^) or T helper cells (CD3^+^CD4^+^).

## **Cytotoxicity Assay**

Antigen-specific cytotoxicity of effector PBMCs before and after immunization, was measured using the GranToxiLux® kit (OncoImmunin Inc., Gaithersburg, MD). Autologous target PBMCs were pulsed with vehicle (no peptide), actin, or HPV-specific peptides for 45 minutes at 37°C in a 5% carbon dioxide (CO_2_) incubator; after which, they were labelled with a fluorescent dye. Effector PBMCs were mixed with the fluorescently labeled autologous targets at an effector: target cell (E:T) ratio of 15:1 and incubated for 1 hour at 37°C in a 5% CO_2_ incubator. The background killing was established in targets in the absence of effectors (target only). Cytotoxicity was determined as the percent (%) of target cells expressing cleaved granzyme B substrates as detected by multi-color flow cytometry.

**ELISA**

Direct ELISA method was developed to assess the immune reactivity of monkey serum against the antigens contained within the fusion E7/E6* protein. Briefly, ELISA plates were coated with HPV-16 E6* and E7* separately in concentration 1 mg/mL and incubated at 4^0^C for 16 hours. Then plates were washed with PBS/0.05% Tween20 and blocked with PBS/0.05% Tween20/1%BSA for 1h at ambient temperature and then washed again. NHP serum samples were titrated 4-fold from 1:200 to 1:10,000,000 and added to E6- and E7-coated plates, then incubated at ambient temperature with shaking (500 rpm, 2h). Bound immunoglobulin was detected with HRP-conjugated anti-human Fc antibody (1:10,000) incubated at ambient temperature with shaking (500 rpm, 1h). After a final wash, a TMB substrate was added and colorimetric reaction was allowed to proceed for 20 minutes. The reaction was stopped with 2N sulfuric acid, and plates read at 450 and 540 nm in a spectrophotometer. Final values are determined as OD differential between 450 nm and 540 nm reads. The titration curves for each sample were plotted using a 4-parameter semi-log fit, and EC50 determined.

**References**

1. [Zimmermann H](https://www-ncbi-nlm-nih-gov.ezp-prod1.hul.harvard.edu/pubmed/?term=Zimmermann%20H%5BAuthor%5D&cauthor=true&cauthor_uid=10400710), [Degenkolbe R](https://www-ncbi-nlm-nih-gov.ezp-prod1.hul.harvard.edu/pubmed/?term=Degenkolbe%20R%5BAuthor%5D&cauthor=true&cauthor_uid=10400710), [Bernard HU](https://www-ncbi-nlm-nih-gov.ezp-prod1.hul.harvard.edu/pubmed/?term=Bernard%20HU%5BAuthor%5D&cauthor=true&cauthor_uid=10400710), [O'Connor MJ](https://www-ncbi-nlm-nih-gov.ezp-prod1.hul.harvard.edu/pubmed/?term=O%27Connor%20MJ%5BAuthor%5D&cauthor=true&cauthor_uid=10400710). The human papillomavirus type 16 E6 oncoprotein can down-regulate p53 activity by targeting the transcriptional coactivator CBP/p300. J [Virol.](https://www-ncbi-nlm-nih-gov.ezp-prod1.hul.harvard.edu/pubmed/10400710) 1999; 73: 6209-19.
2. Zanier K, ould M'hamed ould Sidi A, Boulade-Ladame C, Rybin V, Chappelle A, Atkinson A, et al. Solution structure analysis of the HPV16 E6 oncoprotein reveals a self-association mechanism required for E6-mediated degradation of p53. Structure. 2012; 20: 604-617.
3. Zanier K, Charbonnier S, Sidi AO, McEwen AG, Ferrario MG, [Poussin-Courmontagne P](https://www.ncbi.nlm.nih.gov/pubmed/?term=Poussin-Courmontagne%20P%5BAuthor%5D&cauthor=true&cauthor_uid=23393263), et al. Structural basis for hijacking of cellular LxxLL motifs by papillomavirus E6 oncoproteins. Science. 2013; 339: 694-698.
4. Stahl-Hennig C, Eisenblätter M, Jasny E, Rzehak T, Tenner-Racz K, [Trumpfheller C](https://www.ncbi.nlm.nih.gov/pubmed/?term=Trumpfheller%20C%5BAuthor%5D&cauthor=true&cauthor_uid=19360120), et al. Synthetic double-stranded RNAs are adjuvants for the induction of T helper 1 and humoral immune responses to human papillomavirus in rhesus macaques. PLoS Pathog. 2009; 5:e1000373.
