# Supplementary material for: Synthetic vaccine particles for durable cytolytic T lymphocyte responses and anti-tumor immunotherapy
Source: PLoS One. 2018 Jun 1;13(6):e0197694. doi: 10.1371/journal.pone.0197694 (PMC5983463; doi:10.1371/journal.pone.0197694)
Supplement: S1 Fig — Animals (3–6 per time-point) were injected with SVP[OVA]-PLGA combined with SVP[R848] and CTL activity measured in vivo at times indicated (percentage of cells killed is shown for each time-point). (DOCX) [file pone.0197694.s002.docx]

**Supporting information Figure S1. Long-term induction of antigen-specific cytotoxicity by SVP-PLGA.** Animals (3-6 per time-point) were injected with SVP[OVA]-PLGA combined with SVP[R848] and CTL activity measured in vivo at times indicated (percentage of cells killed is shown for each time-point).
