# Supplementary material for: Synthetic vaccine particles for durable cytolytic T lymphocyte responses and anti-tumor immunotherapy
Source: PLoS One. 2018 Jun 1;13(6):e0197694. doi: 10.1371/journal.pone.0197694 (PMC5983463; doi:10.1371/journal.pone.0197694)
Supplement: S2 Fig — N1, N2 –irrelevant TC- 1 tumor tissues. Samples 1–3, 6, 7 and 11 –mice treated with SVP[Empty], tumor tissues taken at sacrifice on days 18 to 21; samples 4, 5, 8–10 and 12 –mice treated with SVP[OVA]-PLGA and SVP[R848], tumor tissues taken at sacrifice on days 24 to 32. A, B–amplification with two different primer pairs located within OVA gene (expected sizes 814 and 424 bp, correspondingly), C– β-globin-specific primers, M– 100 bp ladder MW markers (500-bp and 1000-bp fragments indicated). (DOCX) [file pone.0197694.s003.docx]

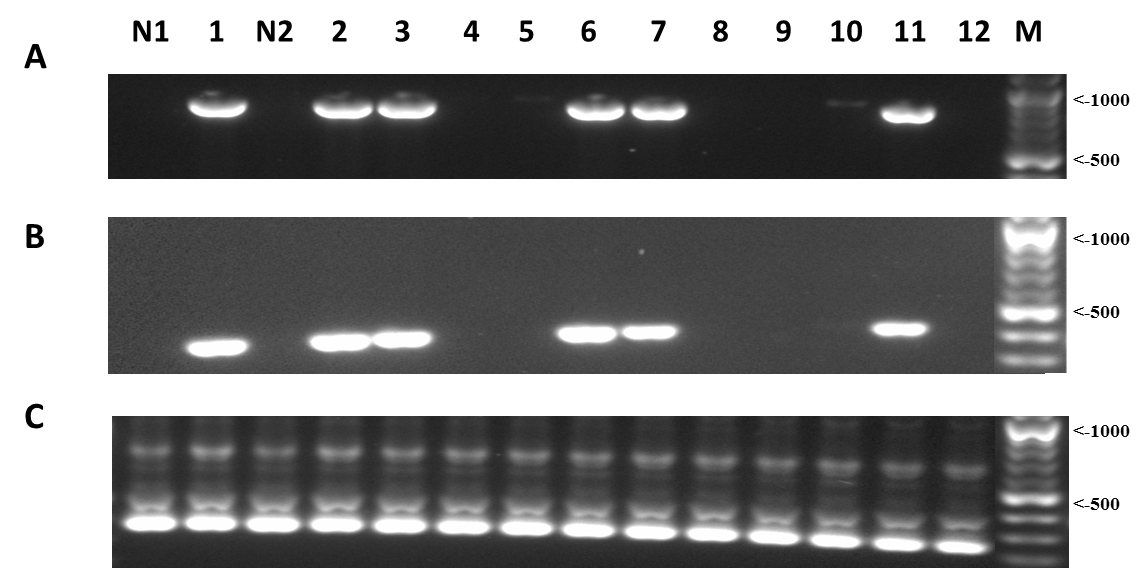


**Supporting information Figure S2. PCR amplification of OVA-specific DNA sequences from EG.7-OVA tumors from SVP-treated mice.** N1, N2 – irrelevant TC- 1 tumor tissues. Samples 1-3, 6, 7 and 11 – mice treated with SVP[Empty], tumor tissues taken at sacrifice on days 18 to 21; samples 4, 5, 8-10 and 12 – mice treated with SVP[OVA]-PLGA and SVP[R848], tumor tissues taken at sacrifice on days 24 to 32. **A**, **B** – amplification with two different primer pairs located within OVA gene (expected sizes 814 and 424 bp, correspondingly), **C** – β-globin-specific primers, M – 100 bp ladder MW markers (500-bp and 1000-bp fragments indicated).
