# Supplementary material for: Synthetic vaccine particles for durable cytolytic T lymphocyte responses and anti-tumor immunotherapy
Source: PLoS One. 2018 Jun 1;13(6):e0197694. doi: 10.1371/journal.pone.0197694 (PMC5983463; doi:10.1371/journal.pone.0197694)
Supplement: S3 Fig — Mice were injected with SVP[OVA]-PLA or SVP[OVA]-PLGA containing Cy5-labeled polymer and DQ-labeled OVA. At 2–48 hours draining LN were taken, processed and stained with surface marker antibodies and analyzed by FACS. Shares of cell populations emitting Cy5- and DQ-based fluorescence (top and bottom rows, correspondingly) are shown for B cells (A) and different DC subtypes (B-E). (DOCX) [file pone.0197694.s004.docx]

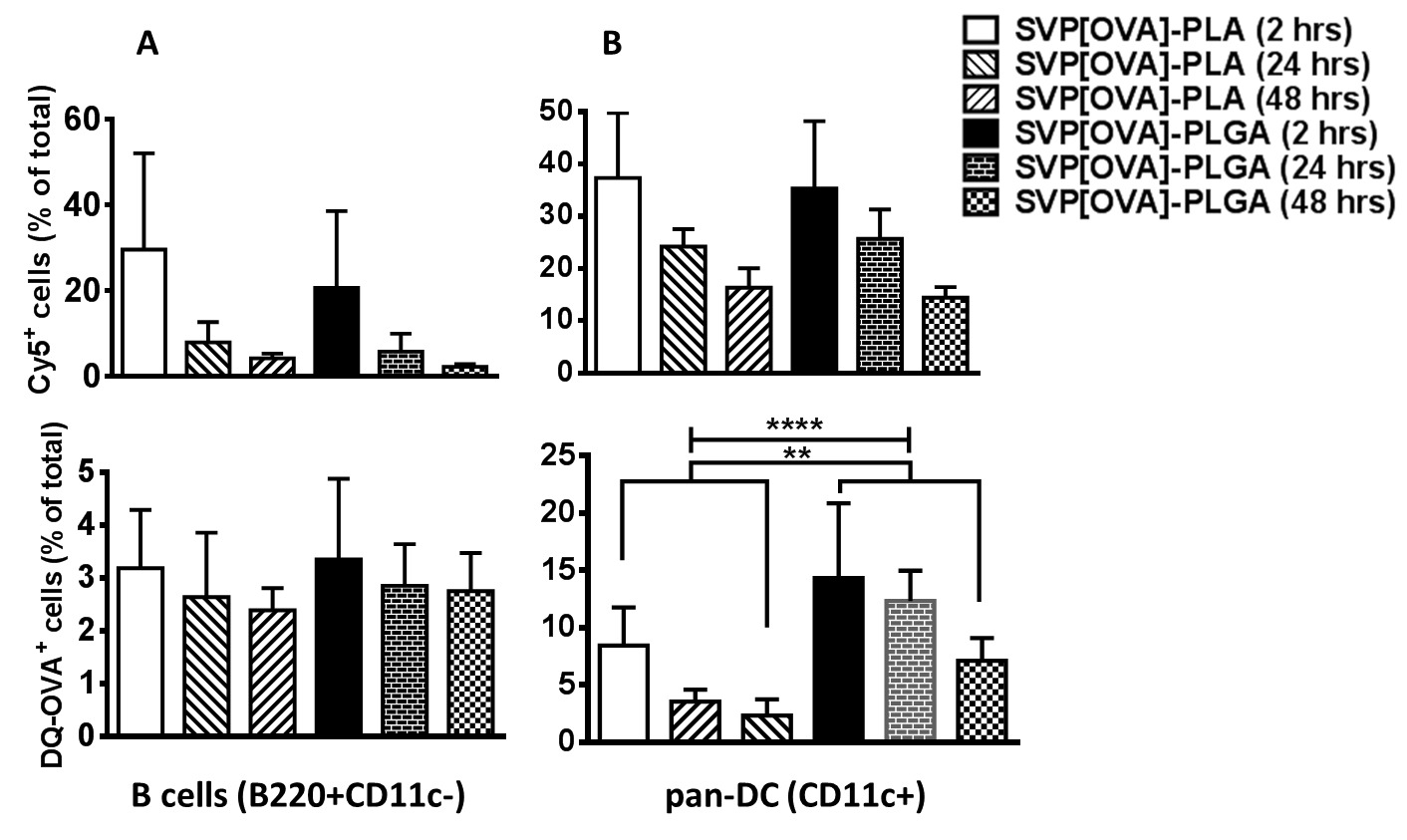


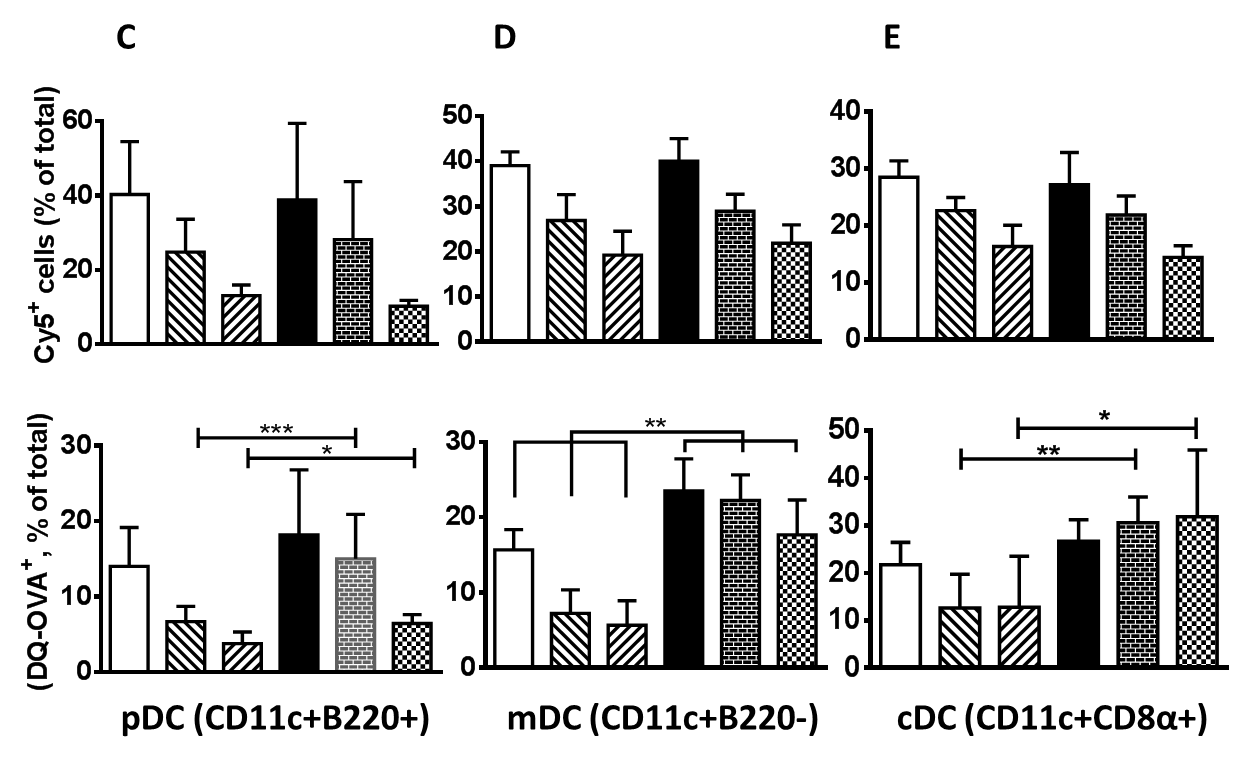


**Supporting information Figure S3. Local SVP trafficking and antigen processing.** Mice were injected with SVP[OVA]-PLA or SVP[OVA]-PLGA containing Cy5-labeled polymer and DQ-labeled OVA. At 2-48 hours draining LN were taken, processed and stained with surface marker antibodies and analyzed by FACS. Shares of cell populations emitting Cy5- and DQ-based fluorescence (top and bottom rows, correspondingly) are shown for B cells (**A**) and different DC subtypes (**B-E**).
