# Supplementary material for: Synthetic vaccine particles for durable cytolytic T lymphocyte responses and anti-tumor immunotherapy
Source: PLoS One. 2018 Jun 1;13(6):e0197694. doi: 10.1371/journal.pone.0197694 (PMC5983463; doi:10.1371/journal.pone.0197694)
Supplement: S4 Fig — A, B. Mice were immunized at d0, 21 and 42 with SVP[OVA] + SVP[R848] (A) or with SVP[OVA] + SVP[CpG] with free and SVP-entrapped PS- and PO-forms of CpG ODN 7909 (B). Anti-OVA IgG titers were measured at d122 and d721 (A) or at d33 and d372 (B). C. Late boost of pre-immunized mice. Mice described in A were split into three groups and boosted at d742 with SVP[R848] + SVP[OVA] at different doses of OVA (shown at X-axis) and titers measured on d721, 754, and 768. D, E. Antigen-specific IFN-γ induction in PBMC from long-term immunized animals. D. Mice were immunized with SVP[R848] + SVP[OVA]-PLGA; direct ex vivo ELISPOT with OP.I.257 peptide was run within 200–400 days after the last SVP immunization. E. Mice were immunized (3 times; d0, 21, 42)) with SVP[OVA]-PLGA combined with SVP-entrapped PS-/PO-CpG or free PS-CpG; direct ex vivo ELISPOT was run at 330d after the last SVP immunization. (DOCX) [file pone.0197694.s005.docx]

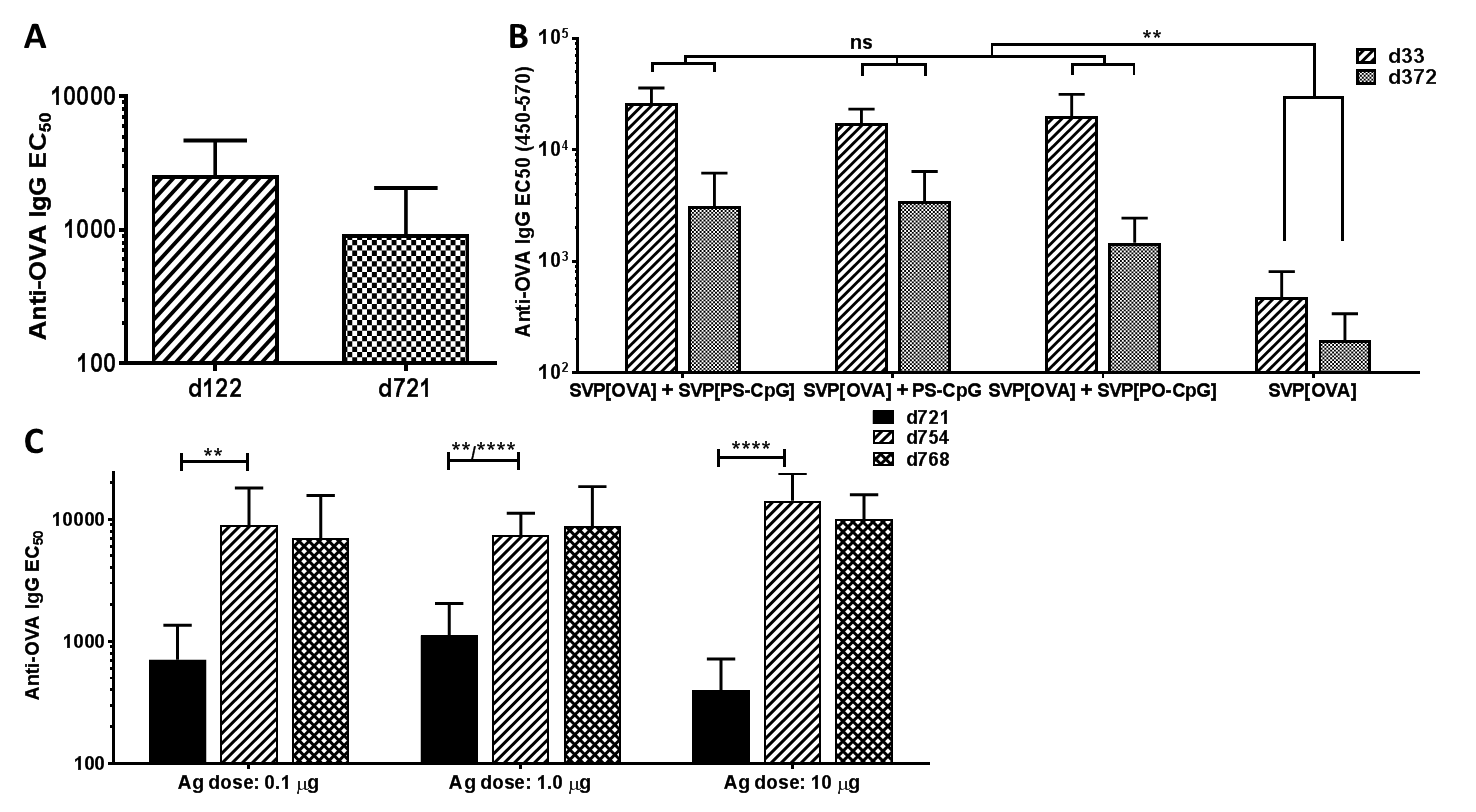


**
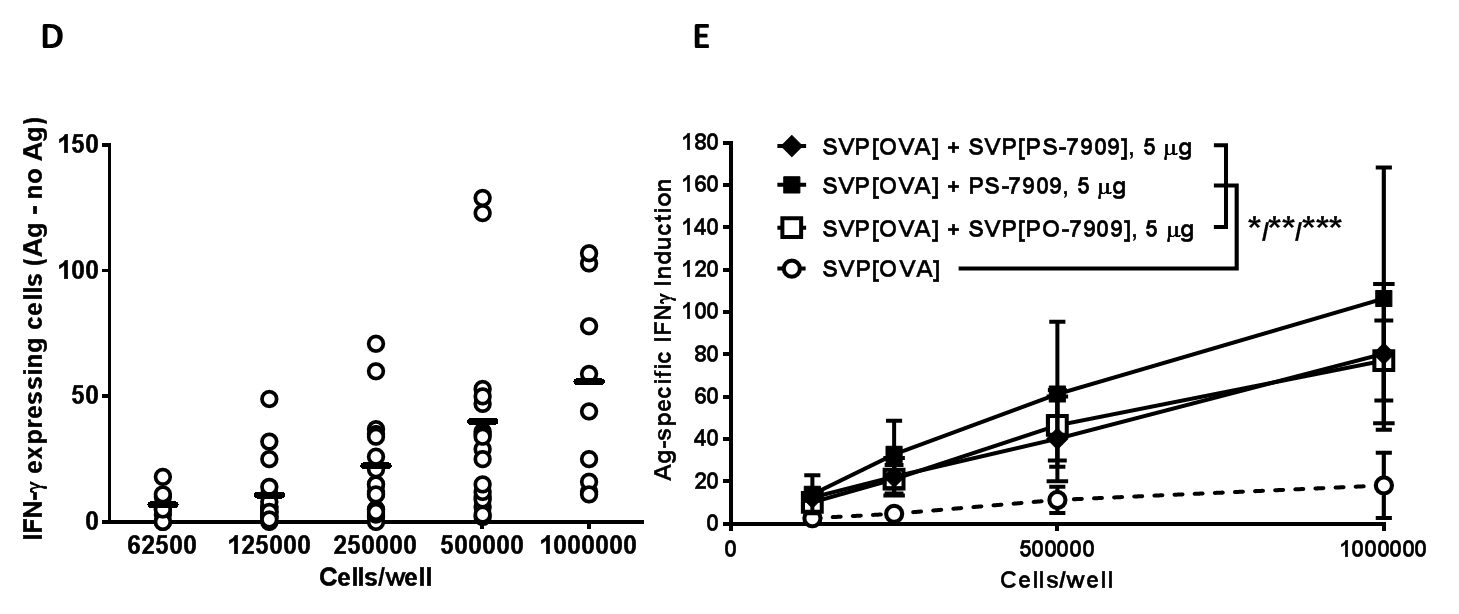
**

**Supporting information Figure S4. SVP induce long-term immune memory. A, B.** Mice were immunized at d0, 21 and 42 with SVP[OVA] + SVP[R848] (**A**) or with SVP[OVA] + SVP[CpG] with free and SVP-entrapped PS- and PO-forms of CpG ODN 7909 (**B**). Anti-OVA IgG titers were measured at d122 and d721 (**A**) or at d33 and d372 (**B**). **C.** Late boost of pre-immunized mice. Mice described in **A** were split into three groups and boosted at d742 with SVP[R848] + SVP[OVA] at different doses of OVA (shown at X-axis) and titers measured on d721, 754, and 768. **D, E.** Antigen-specific IFN-γ induction in PBMC from long-term immunized animals. **D.** Mice were immunized with SVP[R848] + SVP[OVA]-PLGA; direct ex vivo ELISPOT with OP.I.257 peptide was run within 200-400 days after the last SVP immunization. **E.** Mice were immunized (3 times; d0, 21, 42)) with SVP[OVA]-PLGA combined with SVP-entrapped PS-/PO-CpG or free PS-CpG; direct ex vivo ELISPOT was run at 330d after the last SVP immunization.
