# Supplementary material for: Synthetic vaccine particles for durable cytolytic T lymphocyte responses and anti-tumor immunotherapy
Source: PLoS One. 2018 Jun 1;13(6):e0197694. doi: 10.1371/journal.pone.0197694 (PMC5983463; doi:10.1371/journal.pone.0197694)
Supplement: S5 Fig — SVP-entrapped PO- or free CpG PS-1826 injections were carried out on days 0, 4, 11 and 18 (A) or days 0, 21 and 42 (B). Footpad thickness was measured daily. Means with SD are shown. (DOCX) [file pone.0197694.s006.docx]

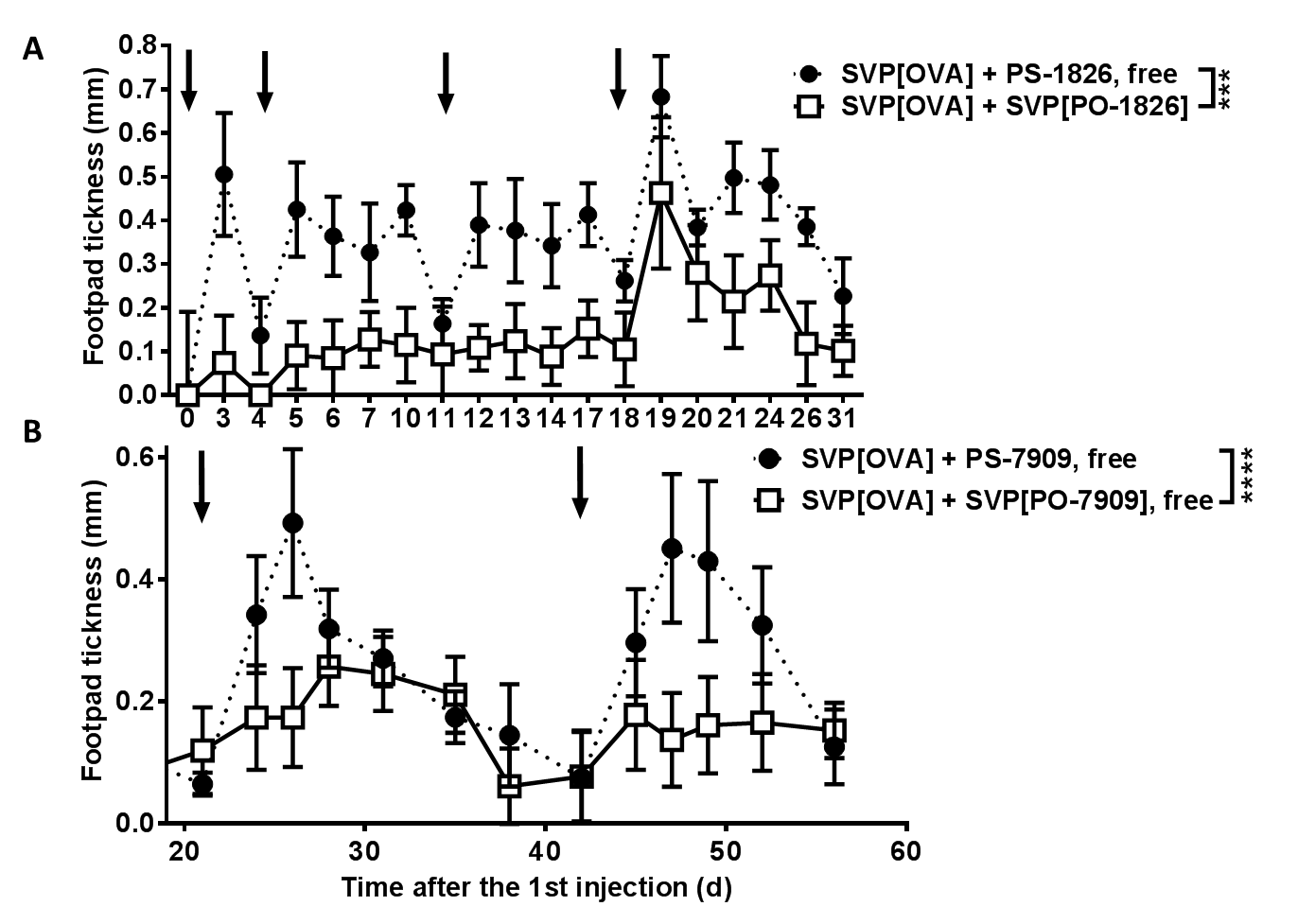


**Supporting information Figure S5. Decrease of local inflammation after injections of SVP[PO-CpG].** SVP-entrapped PO- or free CpG PS-1826 injections were carried out on days 0, 4, 11 and 18 (**A**) or days 0, 21 and 42 (**B**). Footpad thickness was measured daily. Means with SD are shown.
