# Supplementary material for: Synthetic vaccine particles for durable cytolytic T lymphocyte responses and anti-tumor immunotherapy
Source: PLoS One. 2018 Jun 1;13(6):e0197694. doi: 10.1371/journal.pone.0197694 (PMC5983463; doi:10.1371/journal.pone.0197694)
Supplement: S6 Fig — A, B. Inhibition of TC-1 lung seeding by SVP treatment. TC-1 cells were injected i.v., mice were treated at d3, 7, 14 and 21 and lungs harvested on d32. Metastatic counts (A) and lung appearance (B) for SVP- and mock-treated groups are shown with arrows pointing at typical pronounced metastases in lungs from all the untreated mice. C. Treatment of B16-F10 tumors by SVP[Trp2] combined with R848 or CpG ODN PO-2395. Survival proportions are shown. Treatments administered on days 3, 7, 14 and 21 (indicated by arrows; 10–15 mice/group). **–p < 0.01, *** p<0.001, **** p < 0.0001. (DOCX) [file pone.0197694.s007.docx]

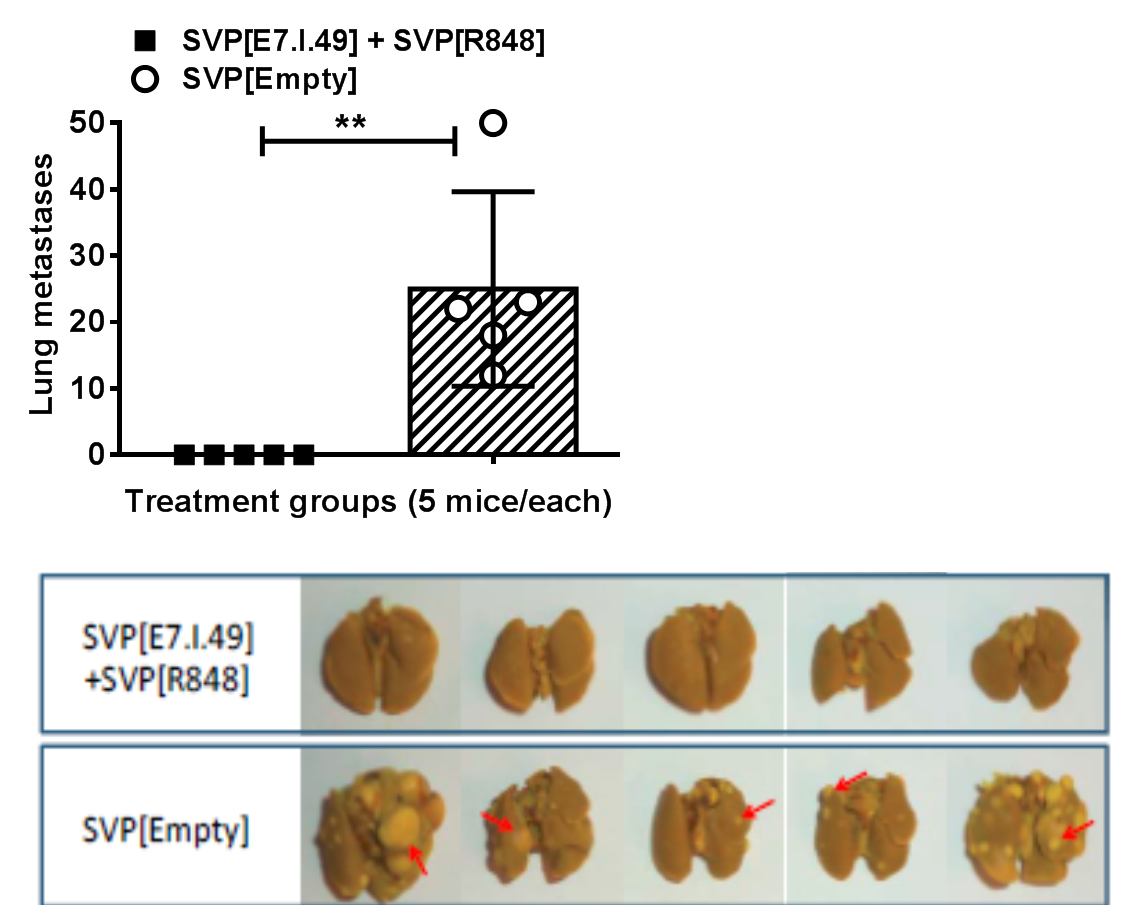


**A**

**B**

**C**

*
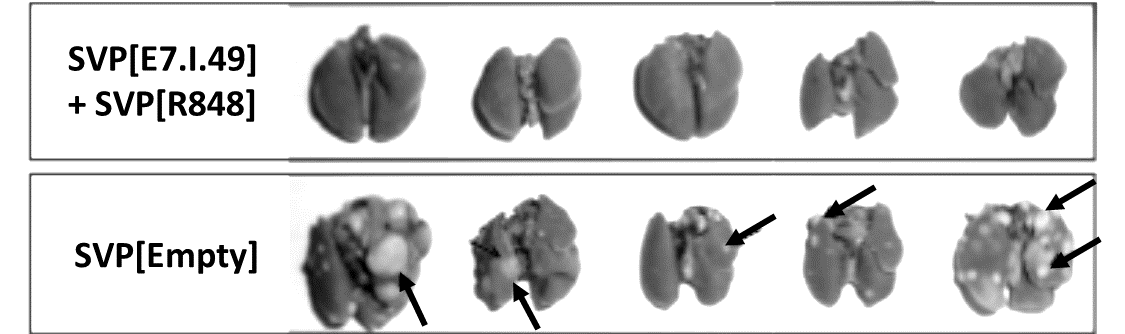
*

**Supporting information Figure S6. Anti-tumor activity of SVP. A, B.** Inhibition of TC-1 lung seeding by SVP treatment. TC-1 cells were injected i.v., mice were treated at d3, 7, 14 and 21 and lungs harvested on d32. Metastatic counts (**A**) and lung appearance (**B**) for SVP- and mock-treated groups are shown with arrows pointing at typical pronounced metastases in lungs from all the untreated mice. **C.** Treatment of B16-F10 tumors by SVP[Trp2] combined with R848 or CpG ODN PO-2395. Survival proportions are shown**.** Treatments administered on days 3, 7, 14 and 21 (indicated by arrows; 10-15 mice/group). ** – p < 0.01, *** p<0.001, **** p < 0.0001.
