# Supplementary material for: Synthetic vaccine particles for durable cytolytic T lymphocyte responses and anti-tumor immunotherapy
Source: PLoS One. 2018 Jun 1;13(6):e0197694. doi: 10.1371/journal.pone.0197694 (PMC5983463; doi:10.1371/journal.pone.0197694)
Supplement: S7 Fig — Induction of E7-specific antibodies by SVP[E7*] and SVP[E7*E6*] (co-injected with SVP[R848]; 5 mice/group). * p <0.05; **p<0.01. (DOCX) [file pone.0197694.s008.docx]

**Supporting information Figure S7. Immunogenicity of SVP-entrapped HPV-16 antigens.** Induction of E7-specific antibodies by SVP[E7*] and SVP[E7*E6*] (co-injected with SVP[R848]; 5 mice/group). * p <0.05; **p<0.01.
