# Supplementary material for: Synthetic vaccine particles for durable cytolytic T lymphocyte responses and anti-tumor immunotherapy
Source: PLoS One. 2018 Jun 1;13(6):e0197694. doi: 10.1371/journal.pone.0197694 (PMC5983463; doi:10.1371/journal.pone.0197694)
Supplement: S8 Fig — Mice were inoculated with either SVP[E7/E6*], SVP[E7/E6*] combined with SVP[poly(I:C)] or admixed with free poly(I:C) and bled at times indicated. The same amount of free or SVP-encapsulated poly(I:C) was used for each group (5 μg). Serum samples were collected at the times indicated and analyzed for individual cytokines by ELISA. Average cytokine concentration is shown (three samples per group per each time-point). A–TNF-α, B–IL-6, C–MCP-1. (DOCX) [file pone.0197694.s009.docx]

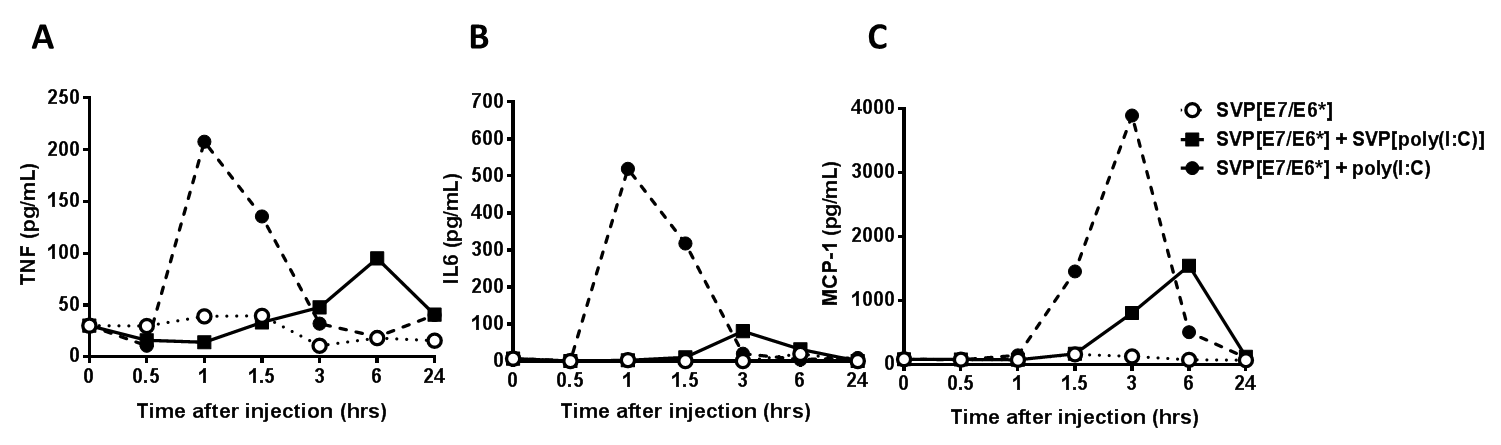


**Supporting information Figure S8.** Systemic cytokine induction after subcutaneous inoculation of free, but not SVP-encapsulated TLR3 agonist poly(I:C). Mice were inoculated with either SVP[E7/E6*], SVP[E7/E6*] combined with SVP[poly(I:C)] or admixed with free poly(I:C) and bled at times indicated. The same amount of free or SVP-encapsulated poly(I:C) was used for each group (5 µg). Serum samples were collected at the times indicated and analyzed for individual cytokines by ELISA. Average cytokine concentration is shown (three samples per group per each time-point). **A** – TNF-α, **B** – IL-6, **C** – MCP-1.
