# Supplementary material for: Synthetic vaccine particles for durable cytolytic T lymphocyte responses and anti-tumor immunotherapy
Source: PLoS One. 2018 Jun 1;13(6):e0197694. doi: 10.1371/journal.pone.0197694 (PMC5983463; doi:10.1371/journal.pone.0197694)
Supplement: S9 Fig — A. Immune memory in SVP-treated B16 survivors from experiments described in Fig 5I. Trp2-specific IFN-γ production in splenocytes of surviving mice (taken on days 104–171 after initial inoculation or days 83–150 after the final SVP treatment) was measured after overnight stimulation. Numbers of mice in each group is shown in parentheses. B. SVP[E7.I.49] and SVP[R848] were injected on days 14, 17, 24 and 31 after TC-1 inoculation either alone or combined with antibody to PD-L1 or isotype control administered on days 18, 21 and 25 (7–8 mice/group). Overall survival is shown (* p<0.05; ** p <0.01). (DOCX) [file pone.0197694.s010.docx]

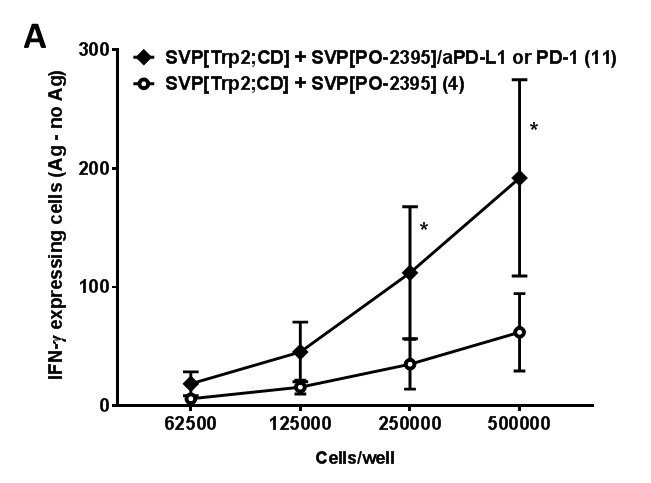

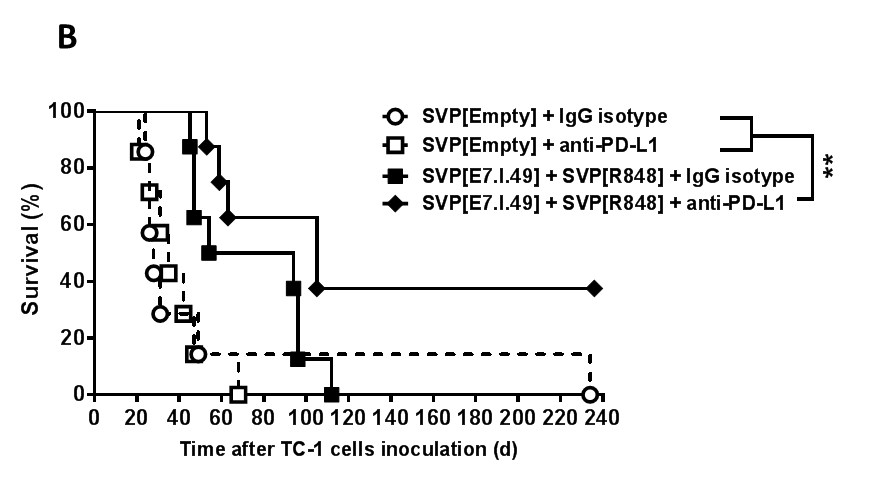


**Supporting information Figure S9. Synergy of SVP and immune checkpoint inhibitors leads to higher survival and immune memory. A.** Immune memory in SVP-treated B16 survivors from experiments described in **Fig. 5I**. Trp2-specific IFN-γ production in splenocytes of surviving mice (taken on days 104-171 after initial inoculation or days 83-150 after the final SVP treatment) was measured after overnight stimulation. Numbers of mice in each group is shown in parentheses. **B.** SVP[E7.I.49] and SVP[R848] were injected on days 14, 17, 24 and 31 after TC-1 inoculation either alone or combined with antibody to PD-L1 or isotype control administered on days 18, 21 and 25 (7-8 mice/group). Overall survival is shown (* p<0.05; ** p <0.01).
