# Supplementary material for: Synthetic vaccine particles for durable cytolytic T lymphocyte responses and anti-tumor immunotherapy
Source: PLoS One. 2018 Jun 1;13(6):e0197694. doi: 10.1371/journal.pone.0197694 (PMC5983463; doi:10.1371/journal.pone.0197694)
Supplement: S10 Fig — Adjuvant and E7/E6* doses specified per Table 1 (high dose– 1, low dose– 2), data grouped in columns per adjuvant used (indicated on top of each set); peptide pools used for PBMC stimulation are shown. Y-axis scale for R848 is 8 times smaller than for CpG and poly(I:C) with the latter two being equal (with the exception of low doses of both E7/E6* and an adjuvant). (DOCX) [file pone.0197694.s011.docx]

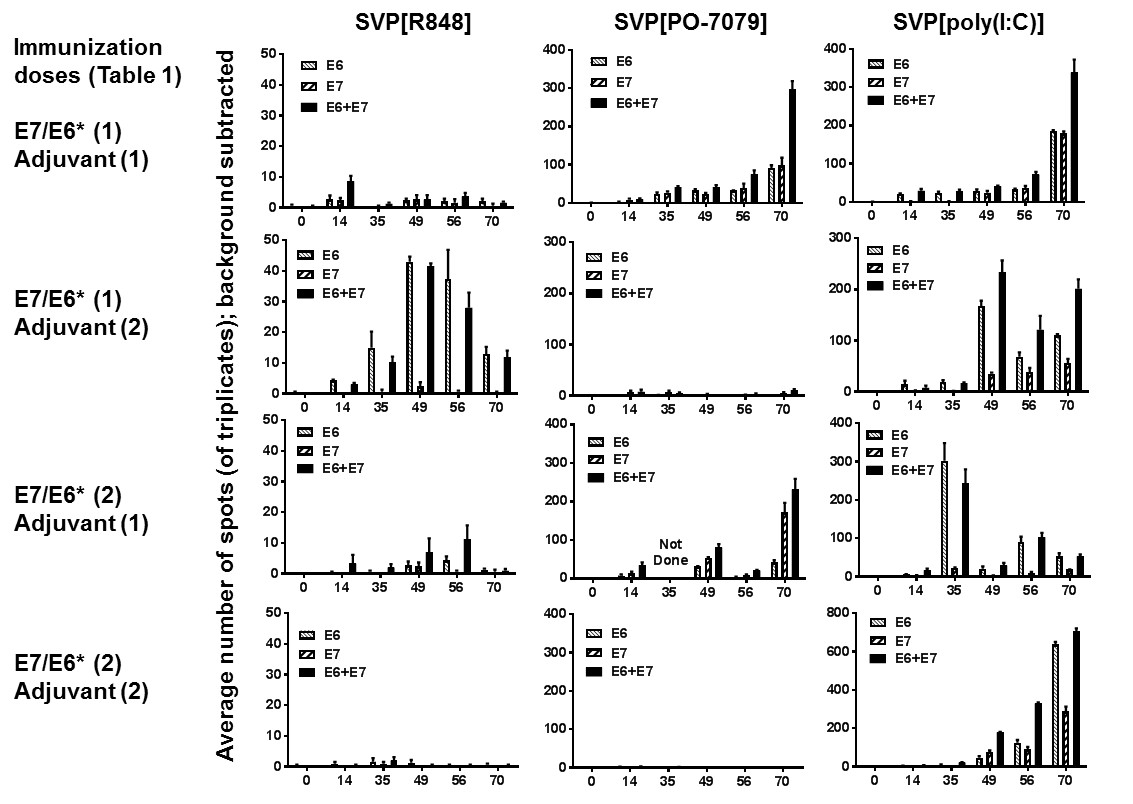


**Supporting information Figure S10.** IFNγ ELISPOT in monkey PBMC; individual graphs. Adjuvant and E7/E6* doses specified per Table 1 (high dose – 1, low dose – 2), data grouped in columns per adjuvant used (indicated on top of each set); peptide pools used for PBMC stimulation are shown. Y-axis scale for R848 is 8 times smaller than for CpG and poly(I:C) with the latter two being equal (with the exception of low doses of both E7/E6* and an adjuvant).
