# Supplementary material for: Synthetic vaccine particles for durable cytolytic T lymphocyte responses and anti-tumor immunotherapy
Source: PLoS One. 2018 Jun 1;13(6):e0197694. doi: 10.1371/journal.pone.0197694 (PMC5983463; doi:10.1371/journal.pone.0197694)
Supplement: S11 Fig — Adjuvant and E7/E6* doses specified per Table 1 (high dose– 1, low dose– 2), data grouped in columns per adjuvant used (indicated on top of each set); peptide pools used are shown. Y-axis scale for all adjuvants is identical with the exception of two graphs for both CpG (high adjuvant, low and high E7/E6*) and poly(I:C) (high adjuvant, low E7/E6* and high E7/E6*, low adjuvant), which are of larger scale. (DOCX) [file pone.0197694.s012.docx]

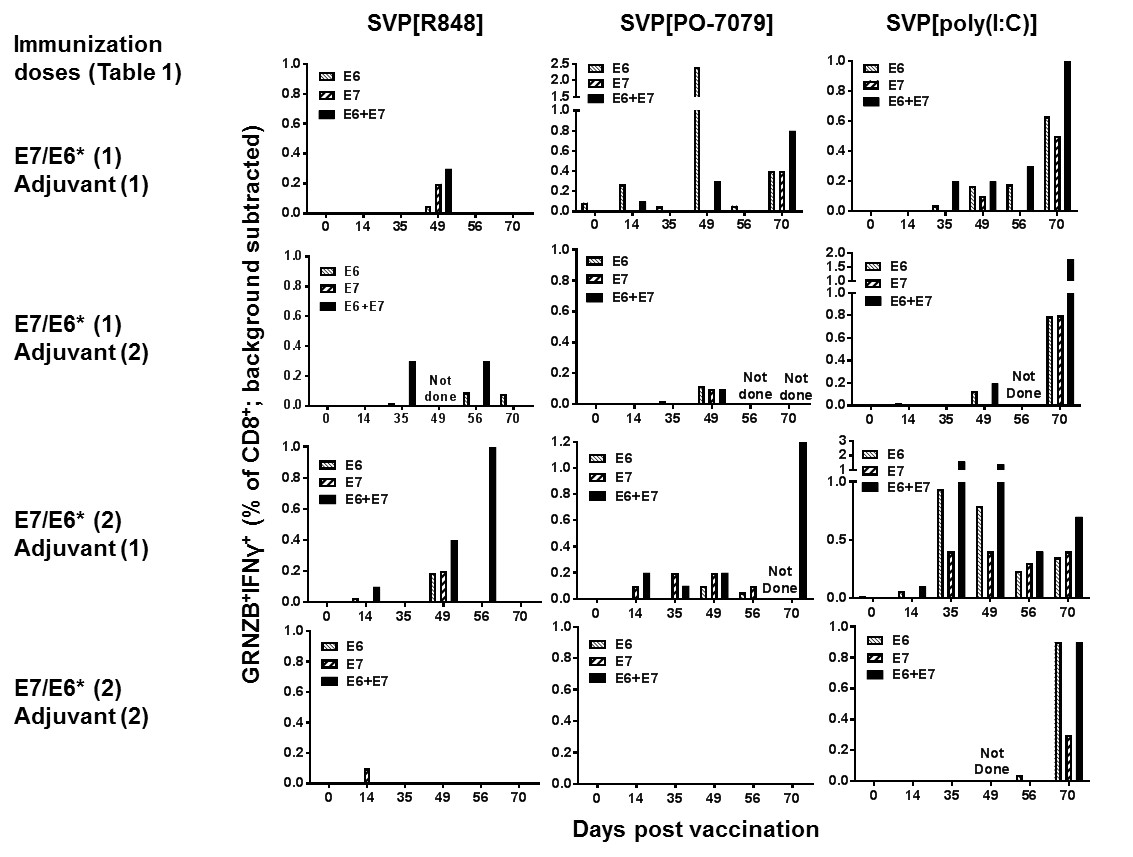


**Supporting information Figure S11.** Granzyme B^+^IFNγ^+^ fractions from total CD8^+^ monkey PBMC after peptide stimulation; individual graphs. Adjuvant and E7/E6* doses specified per Table 1 (high dose – 1, low dose – 2), data grouped in columns per adjuvant used (indicated on top of each set); peptide pools used are shown. Y-axis scale for all adjuvants is identical with the exception of two graphs for both CpG (high adjuvant, low and high E7/E6*) and poly(I:C) (high adjuvant, low E7/E6* and high E7/E6*, low adjuvant), which are of larger scale.
